# Aerosol-based disinfection protocol for aircraft: a solution for the conflict between material compatibility and antimicrobial efficacy

**DOI:** 10.1128/msphere.00542-26

**Published:** 2026-08-31

**Authors:** Sandro Klafack, Jessica Kohs, Karla A. Schwenke, Paul Siller, Janina Reißner, Holger Freese, Alina Below, Tim Lübcke, Stefan Mehler, Bernd Albert, Heiko Barth, Sabine Hoelterhoff, Mathias Streitz, Marcell Engel, Jan Schinköthe, Hendrik A. Scheinemann, Marc Thanheiser, Uwe Rösler, Jens Teifke, Sven Reiche

**Affiliations:** 1 Department of Experimental Animal Facilities and Biorisk Management (ATB), Friedrich-Loeffler-Institut, Federal Research Institute for Animal Health 39023, Greifswald–Insel Riems, Germany; 2 Division Hospital Hygiene, Infection Prevention and Control, Department of Infectious Diseases, Robert Koch Institute, Berlin, Germany; 3 Institute for Animal Hygiene and Environmental Health, Freie Universität Berlin 9166, Berlin, Germany; 4 Lufthansa Technik AG, Hamburg, Germany; 5 Lufthansa Technik AG, Frankfurt am Main, Germany; 6 AKUT SOS CLEAN GmbH (SOS), Frankfurt am Main, Germany; 7 Institute of Veterinary Pathology, Faculty of Veterinary Medicine, Leipzig University 9180, Leipzig, Germany; University of Nebraska Medical Center College of Medicine, Omaha, Nebraska, USA

**Keywords:** dry fog, disinfection, peroxyacetic acid, aircraft, aerosol-based

## Abstract

Every year, billions of passengers, pets, and livestock are transported by air, which increases the risk of a global spread of pathogens like severe acute respiratory syndrome coronavirus 1 and 2, H1N1 influenza, the Ebola virus, and foot-and-mouth disease virus. However, due to its complexity and high safety standards, aircraft decontamination is limited to manual surface disinfection, and an approved method for disinfecting otherwise inaccessible areas is still unavailable. To address this issue, we present the development and validation of an effective room disinfection method utilizing low concentrations of aerosolized peroxyacetic acid (aPAA), stabilized by hydrogen peroxide, known as “dry fog.” Therefore, we determined the minimal effective antimicrobial concentration of aPAA for a wide range of agents, including bacteria, viruses, and bacterial endospores. Then, we applied the developed disinfection protocol to a large panel of relevant materials multiple times in the context of the official material compatibility tests (MCTs) requested by the European Union Aviation Safety Agency (EASA) to demonstrate that its application will not cause a negative effect on the aircraft airworthiness. After successfully passing these complex material compatibility tests, we finally validated the disinfection protocol within a narrow-body aircraft A320 for its antimicrobial efficacy by using the most resistant microorganisms to aPAA among our test organisms, the bacteriophage MS2, and spores of *Geobacillus stearothermophilus*. Thus, we are now able to present the first aerosol-based room disinfection method suitable for aircraft that could easily be adopted for other aircraft types and other means of mass transport as well.

**IMPORTANCE:**

Despite numerous outbreaks in recent years, particularly the Ebola virus epidemic and the severe acute respiratory syndrome coronavirus 2 pandemic, there is still no approved method for disinfecting contaminated aircraft. Due to the highly complex nature of aircraft material and technology, it is difficult to balance the antimicrobial efficacy and material compatibility of the biocides used in a way that meets high safety standards. Here, we demonstrate an aerosol-based disinfection protocol using a mixture of peracetic acid and hydrogen peroxide. This protocol passed all material compatibility tests according to relevant aircraft certification specifications established by the European Union Aviation Safety Agency, as well as the manufacturer qualification test programs, and proved to be highly effective against viruses, bacteria, and even bacterial endospores without compromising the airworthiness of the aircraft. By targeting both contaminated surfaces and objects, as well as airborne pathogen reservoirs, it minimizes the spillover risk of zoonotic agents and prevents the mechanical transmission of high-consequence agricultural pathogens into disease-free geographical regions. This makes it the first suitable semi-automatic airborne disinfection method that can be used in addition to routine manual surface disinfection following contamination resulting from contact with people, animals, or goods infected or contaminated with high-consequence pathogens.

## INTRODUCTION

The transport of people, animals, and goods by rail, road, water, or air continues to increase worldwide as globalization progresses (1). For instance, in 2014, the Lufthansa Group transported not only 106 million passengers but also more than 110 million animals. As demonstrated in the past decades by the epidemics and pandemics of severe acute respiratory syndrome coronavirus 1 and 2 (SARS-CoV-1/-2), Middle East respiratory syndrome coronavirus (MERS-CoV), influenza, and Ebola virus, this poses a risk for the global spread of infectious agents to humans and animals or their vectors within an extremely short time. Also, bacteria such as the multi-resistant pathogens of tuberculosis or Q fever, as well as purely animal pathogens such as African swine fever virus (ASFV) or Foot-and-mouth disease virus (FMDV), exhibit such a potential (2–4). Additionally, the possibility of an intentional spreading of *Bacillus anthracis* spores as a bioterrorist attack is also given. The high passenger density and limited space inside aircraft cabins can facilitate the transmission of airborne and droplet-borne pathogens. Under certain conditions, these environments may contribute to increased transmission rates and the occurrence of superspreading events. Currently, there is neither a validated nor an approved room disinfection method that can be performed in the event of a highly pathogenic agent on board a mass transportation vehicle in Europe. In particular, aircraft are challenging due to their complex interiors equipped with sensitive instruments and a variety of materials that have to comply with the high safety standards in aviation. In Germany, neither the Robert Koch Institute’s (RKI) list of tested and approved procedures in accordance with §18 of the Infection Protection Act (IfSG) nor the disinfection guideline for certain epizootic diseases of the Federal Ministry of Agriculture, Food and Regional Identity (BMLEH) updated by the Friedrich-Loeffler-Institut (FLI) list any fumigation or aerosol-based procedures for use in aircraft or other complex means of mass or goods transport. This also applies to the guidelines of both the World Health Organization (WHO) and the World Organisation for Animal Health (WOAH). The WHO guidelines (5) define a two-row zone according to previous literature (6). If a passenger shows symptoms of a high-consequence infectious disease, only the surfaces of their seat and those in the same and adjacent rows require wet-chemical disinfection. However, a more detailed analysis revealed that 40% of reported transmissions occurred outside the two-row zone due to the natural movements of the potential index passenger, the other passengers, and the cabin crew during the flight (7). From an epidemiological perspective, the traditional “two-row” approach provides only a limited representation of the actual contact dynamics within a pressurized aircraft cabin. Factors such as airflow patterns, interactions with contaminated surfaces in shared areas like lavatories and galleys, and the movement of airborne particles contribute to a complex and dynamic transmission environment. In addition, manual surface cleaning is highly dependent on human performance and may leave areas that are difficult to access insufficiently treated, allowing pathogens to remain on surfaces for extended periods. This persistence can create ongoing exposure risks for cleaning personnel, maintenance workers, and future passengers, potentially contributing to the spread of infections across different regions.

Therefore, it must be assumed that these disinfection requirements are insufficient to prevent the transmission of pathogens to people on subsequent flights or to cleaning and maintenance personnel (4, 8–10). This equally applies to animals transported in the cargo compartment. Therefore, an additional airborne disinfection step is necessary to reach all possibly contaminated areas, even those which are inaccessible to the wet-chemical disinfection of surfaces, to rule out the risk to the population and the environment. Otherwise, the competent authorities represented by the public health officer or official veterinarian would have to prohibit further operation of the means of transport for an indefinite period of time until clearance is declared.

In order to develop new procedures and materials for the maintenance of aircraft, including the use of disinfectants, they must be approved by the European Aviation Safety Agency (EASA) in accordance with the regulations. As part of the Lufthansa Group, Lufthansa Technik AG (LHT) is certified by EASA and authorized for part 145 (11), part 21/G (11), and part 21/J (12) maintenance, production, and design organization. The EASA Part 21/J (12) approval process for the release of the process can be carried out based on material compatibility tests (MCT). Once the whole verification process, including the results of the MCT, has been completed, a Change/Repair Approval Sheet (CRAS) can be issued. Due to the complexity of the procedure, approval is only granted for a specific aircraft type or in some cases, even only for a single aircraft. The CRAS forms the basis under aviation law for the subsequent application of the procedure to an aircraft by EASA Part 145 (11) authorized personnel like aircraft mechanics. In this context, it must be considered whether applying the disinfectant could have a negative effect on the aircraft airworthiness, for example, by causing chemical changes such as corrosion, degradation of the mechanical properties, or flame retardancy of the components (Boeing D6-7127 [13] and D6-17487 [14], AMS 1450 [15], DIN EN ISO 527:2012 [16], DIN EN ISO 2813-15 [17], ASTM-F502 [18], ASTM-F483 [19], ASTM-D471 [20], ASTM-F1110 [21], and Airbus AIMS 09-04-000 [22]). Additionally, it must be determined whether the (repeated) application of the process can influence the optical appearance of the surfaces (e.g., painted, anodized surfaces, plastics, fabrics, leather, and upholstery), including color (fading), general impairment (shinier or duller surfaces), or even embrittlement (cracking) of the decorative surfaces, which would have no effect on the airworthiness (Boeing D6-7127 [13], Airbus AIMS 09-04-000 [22], and ASTM-F502 [18]).

Recent studies have demonstrated that dry fogging with peroxyacetic acid (PAA), which is present as an equilibrium liquid mixture comprising PAA, acetic acid, and water stabilized by a surplus of hydrogen peroxide, is highly effective against a wide spectrum of microorganisms at low concentrations. This makes it material-friendly as well (23, 24). Here, we demonstrate the development and validation of a protocol using low-concentration, aerosolized PAA (aPAA) as a suitable airborne disinfection method to safely decontaminate aircraft or other means of transport from high-consequence pathogens.

## RESULTS

### Biological safety cabinet

#### Determination of the effective microbicidal concentrations

**Fig 1:**
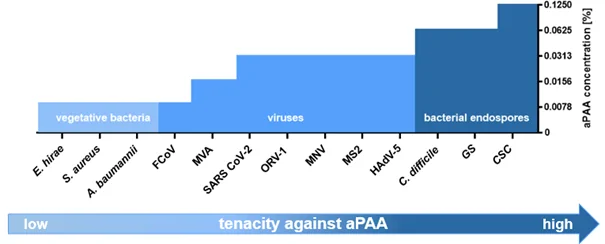
Determination of the effective concentration of aPAA for vegetative bacteria, enveloped and non-enveloped viruses, and bacterial spores when dried on stainless steel carriers. The minimal effective concentrations of aPAA necessary to reach the required reduction are shown (viruses and bacterial endospores >4 log_10_ levels and vegetative bacteria >5 log10 levels; germ carriers were inoculated with *A. baumannii*, *Acinetobacter baumannii; E. hirae*, *Enterococcus hirae; S. aureus*, *Staphylococcus aureus*; FCoV, Feline coronavirus; MVA, Modified-Vaccinia-Ankara-Virus; MNV, murine norovirus; ORV-1, Mammalian orthoreovirus 1; SARS-CoV-2, severe acute respiratory syndrome coronavirus 2; MS2, Escherichia phage MS2; HAdV-5, Human adenovirus 5; *C. difficile*, *Clostridioides difficile* endospores; GS, *Geobacillus stearothermophilus* endospores; CSC, commercial spore carriers coated with GS endospores; *n* ≥ 3).

To determine the minimal effective microbicidal concentration of aPAA, relevant representatives of vegetative bacteria, enveloped and non-enveloped viruses, as well as bacterial spores were dried onto stainless steel carriers and exposed to aPAA concentrations ranging from 0.0078% to 0.125% within a biological safety cabinet (BSC, 1 m^3^) by using the Mini Dry Fog device (Fig. 1).

**Fig 2:**
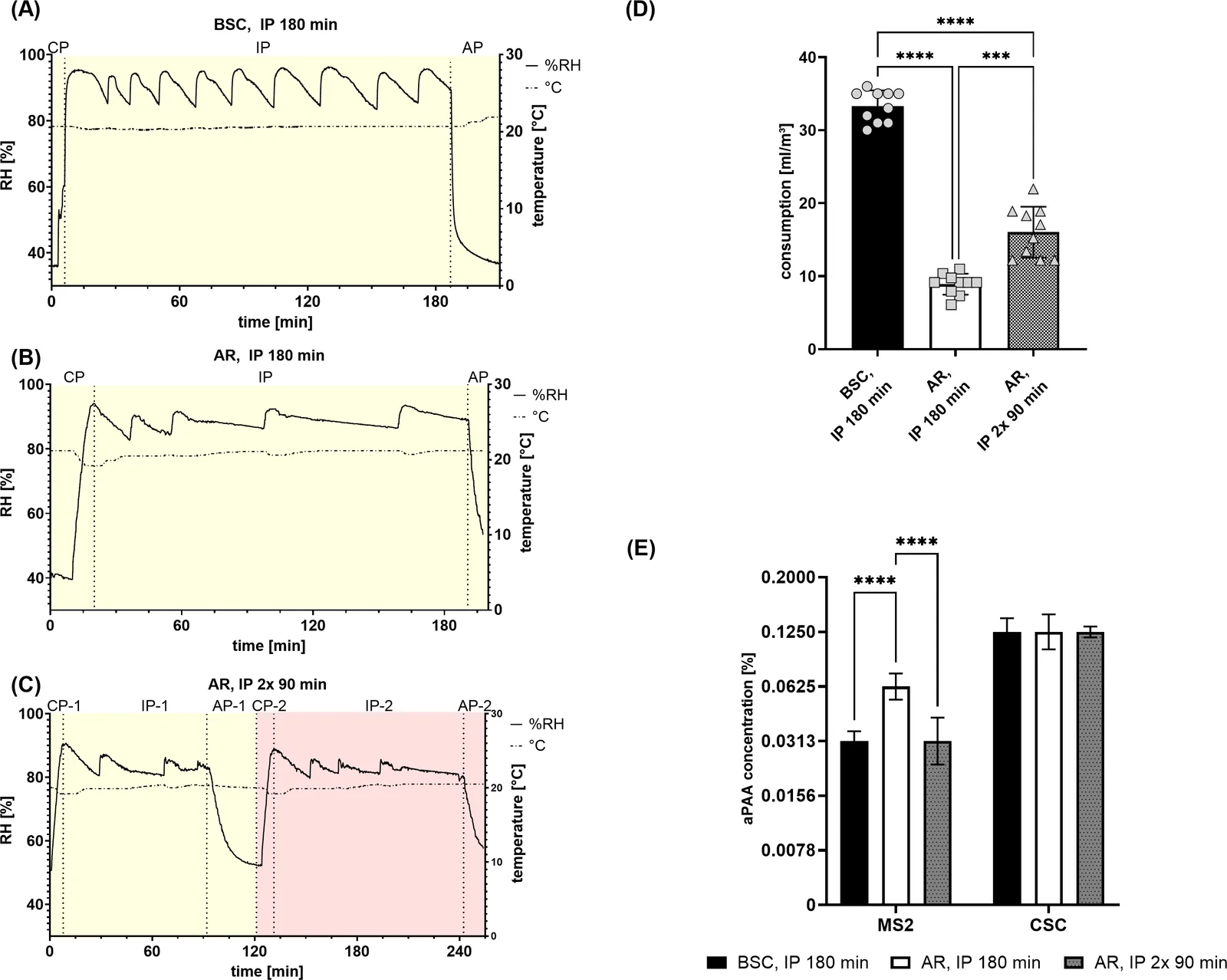
Optimization of the disinfection protocol for larger rooms. (**A**) Climate data of the biological biosafety cabinet (BSC). Representative graphs showing relative humidity (RH) and surface temperature during airborne disinfection in a biological biosafety cabinet with an incubation phase of 180 min (BSC, IP 180 min). The separated conditioning (CP), incubation (IP), and aeration (AP) phases of the disinfection cycle can be identified by following the RH curve. (**B**) Climate data of the animal room (AR). Representative graphs displaying RH and surface temperature during airborne disinfection in an animal room with an incubation phase of 180 min (IP 180 min). (**C**) Climate data of the animal room. Representative graphs displaying the RH and surface temperature during airborne disinfection in an animal room using the modified protocol (IP 2 × 90 min) with two conditioning (CP-1 and CP-2), incubation (IP-1 and IP-2), and aeration phases (AP-1 and AP-2). (**D**) Consumption of PAA at a concentration of 0.125% aPAA. Comparison of the applied PAA consumption per m^3^ for the three different airborne disinfection protocols (0.125% aPAA). A one-way ANOVA following Tukey’s multiple comparisons test was used for statistical comparison. Statistically significant differences are marked with an asterisk: ***P  = 0.0001; ****P < 0.0001. (**E**) Effective aPAA concentration [%] for ≥4 log10 level reduction. Comparison of the minimal necessary aPAA concentration for an efficient inactivation (>4 log10 levels) of MS2 (Escherichia phage MS2) and GS (*Geobacillus stearothermophilus* endospores) using the three different disinfection protocols. A two-way ANOVA following Tukey’s multiple comparisons test was used for statistical comparison. Statistically significant differences are marked with an asterisk: ****P < 0.0001.

The dry fogging process is divided into three phases (Fig. 2A): the conditioning phase (CP) to achieve 85%–90% relative humidity (RH), the incubation phase (IP), and the aeration phase (AP)^5^. Depending on the initial RH (40%–50%), the time needed for the conditioning of the safety cabinet varied slightly. Since the target RH of 85%–90% was reached in <1 min during the CP, it was included in the total incubation time of 180 min. During this, the IP RH was constantly kept between 85%–95%. The following aeration was carried out for a minimum of 30 min to ensure complete removal of the PAA.

Using this protocol, a sufficient reduction of more than 5 log_10_ levels was already achieved with the minimal tested concentration of 0.0078% aPAA for all tested vegetative bacteria (*A. baumannii*, *E. hirae*, and *S. aureus*), while the viruses and bacterial spores showed a higher tolerance against aPAA (Fig. 1; see also Table S1 for more details). The only exception was the enveloped feline coronavirus (FCoV), which could already be sufficiently inactivated by more than 4 log_10_ levels with 0.0078% aPAA. For the other tested enveloped viruses, slightly higher aPAA concentrations were necessary (0.0156% aPAA for Modified Vaccinia virus Ankara [MVA] and 0.0313% aPAA for SARS-CoV-2). The same aPAA concentration of 0.0313% as for SARS-CoV-2 was also effective for all tested non-enveloped viruses (murine norovirus [MNV], mammalian orthoreovirus type 1 [ORV-1], and human mastadenovirus C [HAdV-5]) as well as for the bacteriophage MS2 [MS2]. As expected, the bacterial spores of *Clostridioides difficile* and *Geobacillus stearothermophilus* (GS) showed the highest tolerance against aPAA and were only reduced by >4 log_10_ levels with 0.0625% aPAA. Interestingly, the commercial spore carriers (CSC) coated with *G. stearothermophilus* spores required even 0.125% aPAA for sufficient inactivation.

In order to determine the influence of different carrier materials on the disinfection effect, commercially available polyvinyl chloride (PVC) reference materials (Kömadur ES) and PVC samples produced directly from LHT aircraft interiors were compared with the ISO-standardized stainless-steel carriers for some microorganisms (Table S1). Besides the expected normal biological variations, remarkable differences were only observed for the bacteriophage MS2 when using the PVC Kömadur ES carriers. In this case, significantly higher aPAA concentrations (0.125% aPAA) were required for the requested reduction of more than 4 log_10_ levels compared to the other two carrier materials, which only required 0.0313% aPAA.

#### Optimization of the disinfection protocol for larger rooms and identification of suitable microbiological surrogates for field testing

The minimal effective concentration of aPAA determined in the BSC (1 m^3^) was validated in larger volumes, using two animal rooms of different sizes (21 and 164 m^3^). The disinfectant was aerosolized using the Dry Fog 2-S system with one or four nozzles installed depending on the size of the room.

To adapt and validate the disinfection protocol in the animal rooms, we investigated some of the test organisms with selected concentrations of aPAA (0.0312%, 0.0625%, and 0.125%) based on the prior results. The microorganisms were selected in accordance with the highest tolerance against aPAA and included the non-enveloped viruses ORV-1, MNV, and HAdV-5 (tested only with 0.0625% aPAA) as well as the bacteriophage MS2 and bacterial spores of *C. difficile* and *G. stearothermophilus*. These microorganisms were applied to stainless steel carriers (Fig. S1; Table S2) and subjected to the disinfection protocol (Fig. 2B). Notably, an aPAA concentration of 0.0625% resulted in an effective reduction (>4 log10 levels) for all tested viruses and *C. difficile* spores. As observed in the previous BSC experiments, an aPAA concentration of 0.125% was necessary for sufficient inactivation of the CSC.

Based on these results, the bacteriophage MS2 showed the highest tolerance to aPAA in the group of viruses, and the spores of *G. stearothermophilus* among the bacterial spores. In addition, both microorganisms are non-pathogenic and easy to handle, making them ideal surrogates for the planned field testing. Interestingly, in contrast to the results in the BSC, a reduction of the aPAA concentration to 0.0313% resulted in a significantly lower inactivation efficacy (>2 log_10_ levels) of the bacteriophage MS2 in the larger of the two rooms (Fig. 2E; Tables S1 and S2). A comparison of the PAA consumption per run revealed that three times more PAA per cubic meter was consumed in the BSC than in the animal room to keep the RH constantly above 85% during the incubation time (Fig. 2D). In order to investigate whether the decreased inactivation efficacy in the animal rooms was due to the lower PAA consumption, the room disinfection protocol was modified. The incubation phase of 180 min was divided in half into two separate incubation phases of 90 min each, with an additional aeration and conditioning phase in between (Fig. 2C). This modification resulted in a doubling of consumed PAA (Fig. 2D), and for MS2, a sufficient inactivation was achieved with only 0.0313% aPAA. In contrast, no difference in inactivation efficacy was observed for GS spores (Fig. 2E; Table S2).

### Material compatibility tests

#### Tests with the minimal effective aPAA concentrations

**Fig 3:**
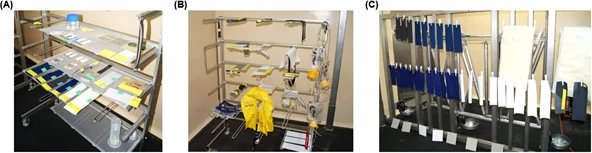
Material samples after 10 dry fogging cycles. (**A** and **B**) Various material samples from the aircraft cabin. (**C**) Fabric, leather, and paint samples.

For the MCT, various relevant aircraft material samples (Table 1) were treated by dry fogging in the larger animal room (164 m^3^) with microbiologically effective aPAA concentrations of 0.0313%, 0.0625%, 0.125%, and, for a few, 1.3% (Fig. 3A and B). Ten dry fogging runs with an incubation time of 180 min each were performed under constant climatic conditions (85%–95% RH, 20°C, Fig. 2B) on consecutive days for each aPAA concentration.

**TABLE 1:** Listed materials used for material compatibility tests with various aPAA concentrations (0.0313%, 0.0625%, 0.125%, and 1.3%)

Material class	Materials
Metals	AL2024 bare/clad^*a*^, AL7075 bare/clad^*a*^, Mg-Alloy^*a*^, TiAl6V4, St AISi321
Sealing material	Sealant(s) (polysulfide-based 2k) applied to aluminum with corrosion protection primer^*a*^
Interior materials	Upholstery fabric^*a*^, textile carpeting^*a*^, curtain fabric^*a*^, various elastomers, PVC hoses for oxygen^*a*^, silicone rubber^*a*^, seat covers, leather and artificial leather, PVC flooring, special materials for work surfaces and wash basins, special wallpaper for aircraft cabins, thermoformed sheets for passenger seats, folding tables for passenger seats, PVC and polycarbonate films, films for insulating mats, insulation mats (40 × 40) cm and for flammability test (12.5 × 23) inch^*a*^, cover for armrests, trim components of aircraft seats, paneling components of aircraft seats, fasteners, aircraft exterior/interior windshield, life jackets, in-flight entertainment PC, laptop, tablet computer, and an SDI Ethernet Encoder Unit^*b*^
Lacquers	Interior paints (solvent and water-based) in light and dark colors, which are painted on aluminum in accordance with the manufacturer’s specifications^*a*^
Adhesive tapes	Velcro and fleece tape^*a*^, various stickers from the aircraft cabin

^*a*^ These materials were also tested with an aPAA concentration of 1.3%.

^*b*^ The Ethernet Encoder Unit was only exposed to 10 cycles of an aPAA concentration of 0.125%.

None of the samples showed any obvious changes when examined immediately after the 10 dry fogging cycles. The IT hardware in the test room (two laptops and a tablet) continuously ran presentations without impairment throughout the entire period. For the MCT with 0.125% aPAA, an Ethernet Encoder Unit was included in the test set additionally, whose function test also passed without findings.

To detect a possible concentration of hydrogen peroxide or PAA in condensed water, which forms in an aircraft cabin during normal operation, a beaker containing water was placed in the room. Only a minimal accumulation of both substances could be detected using test strips. The measured hydrogen peroxide concentration was approximately 2 mg/L, and the PAA concentration was below the detection limit (QUANTOFIX peroxyacetic acid 500).

Subsequently, the material samples were taken to the LHT laboratory for the actual MCT consisting of an optical and functional analysis, flammability test, tensile strength test, sandwich corrosion test, Mg-corrosion test, paint-softening test, and optics/haptics test. Thereby, no traces of corrosion could be detected on the test panels of the sandwich corrosion test and the Mg-corrosion test, as well as the alternatively treated samples. Also, no change or penetration of the active substances could be detected in the samples for sealants, which was confirmed by cross-sectional images. The same was also the case for almost all materials used for the optical and functional analysis, tensile strength test, paint-softening test, and optics/haptics test.

The following few impairments were observed when exposed to the different aPAA concentrations. For all analyzed concentrations, an optical change in brightness was detected on one of the countertop surfaces, which has no relevance for the airworthiness (Fig. 3C). Further tests were carried out in the LHT laboratory to investigate these impairments, simulating intensive exposure to various PAA concentrations. Thereby, the tests were carried out on the countertop surface by applying different dilutions of Lerasept Spezial (0.0625%, 0.125%, and 0.625% PAA) directly by pump spraying to enhance the effect. After drying, the samples were cleaned with water and isopropanol. However, the brightening effect on the surface could not be reproduced in these tests.

Furthermore, a significant (550%) increase up to 26 s of the flame extinguishing time was demonstrated on the seat cover fabrics when exposed to 0.0313% PAA in direct comparison to an untreated reference sample (4 s, flame extinguishing time). This is relevant to airworthiness. Since in an average of at least three samples the flame extinguishing time must not be longer than 15 s, any burning drops that fall off must not burn for longer than 5 s, and the burnt length of the sample must not exceed 203 mm, the test failed for the seat cover fabrics for this tested concentration. Surprisingly, in subsequent tests (same 10× dry fogging simulation) with higher aPAA concentrations (0.0625%, 0.125%, and 1.3%), this behavior could not be reproduced for the fabric seat cover. Also, the curtain fabric exhibited a significant extension of the flame extinguishing time to 18 s (150%) when exposed to 0.0625% aPAA in direct comparison to an untreated reference sample. Hence, the test was not passed for the curtain fabric, either. However, also here this finding could not be observed when treated with the higher aPAA concentrations of 0.125% and 1.3%. In order to further investigate the observed extension of the flame extinguishing time of the seat cover fabrics, additional flammability tests were carried out in the LHT laboratory, simulating intensive exposure to various media (Lerasept Spezial diluted to 0.625% PAA, disinfectant Bacillol AF, or water). For this purpose, the fabric samples were soaked in the media, milled, and after drying for 24 h at room temperature, climate-conditioned for 14 days at 21°C and 50% RH. After conditioning, the flame extinguishing time of all tested samples was below the requested time limit. The afterburning time for the sample soaked in 0.625% PAA was 5.6 s on average, and therefore, it was well below the limit of 15 s (EASA CS25 Appendix F Part I (a) (1) (ii)). The same could be observed for the comparatively treated samples (${water}_{\bar{x}\;flame\;extinguishing}=6.0\;s/{Bacillol\;AF}_{\bar{x}\;flame\;extinguishing}=4.2\;s$).

**Fig 4:**
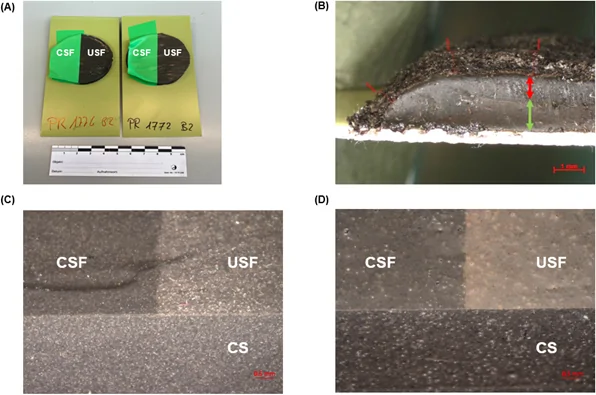
Detailed evaluation of treated sealing components. (**A**) Sealing compounds, with the left side half-masked to protect from treatment and the right side exposed to five consecutive treatments with 1.3% aPAA. (**B**) Sealer exposed to multiple treatments with HP at concentrations ranging from 200 to 400 ppm. Red arrow: damaged part. Green arrow: unaltered part. (**C**) Magnified view of PR1776 from the sealer shown in panel A. (**D**) Magnified view of PR1772 from the sealer shown in panel A. CSF, covered surface; USF, uncovered surface; CS, cross-section.

Even for the 10-fold higher concentration of 1.3% aPAA that was used in order to exploit a possible maximum concentration for use in an aircraft cabin, no impairments were observed except for the sealants. Both tested sealants showed a clear reaction of dissolution, which manifested itself in the form of a slightly sticky surface (Fig. 4A, C and D). However, this dissolution of sealing material on the aircraft structure is critical for airworthiness and therefore not permitted, making the test for this concentration a failure.

### Disinfection of an aircraft by aPAA

**Fig 5:**
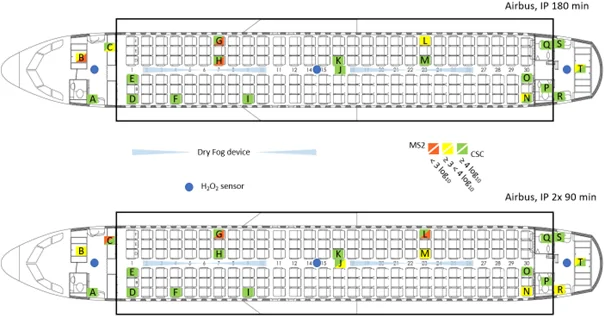
Comparison of the microbicidal efficacy of the two protocols (IP 180 min and IP 2 × 90 min) applied in an Airbus A320. Disinfection protocol with one incubation phase of 180 min (IP 180 min) and disinfection protocol with two incubation phases of 90 min each (IP 2 × 90 min): MS2, Escherichia phage MS2; CSC, commercial spore carriers coated with GS endospores; black frame: passenger area, locations: D, G, I, L, and N under a seat; locations: E, H, M, and O backrest; locations: A, R, and S aircraft door; locations: B, C, and T galley; locations: P and Q lavatories; location: F luggage bin; location: K ceiling; location: J floor; aPAA concentration 0.1250%; *n* = 3 (IP 180 min); *n* = 2 (IP 2 × 90 min).

Following the determination of the minimal effective concentrations of aPAA and their approval as safe for aviation, the procedure was proven in an aircraft. For this purpose, LHT provided an Airbus A320-200 at two distinct time slots, enabling a direct comparison of both established protocols. In the first available time slot, initial tests were conducted using water to assess aerosol dissemination in the cabin, taking into account the tubular geometry of the aircraft, in contrast to the cuboid shape of the animal room, despite the similar volume of 165 m³. This was followed by a complete test run using the selected aPAA concentration of 0.125%. By using a large number of germ carriers coated with bacteriophage MS2 and spores of *G. stearothermophilus* (CSC) in a wide variety of locations, even the most difficult-to-access areas in the passenger compartment (Fig. 5, surrounded by the marked black square) could be identified. Thus, the number of germ carriers required could then be reduced by focusing on 14 critical locations (D–Q) for all subsequent disinfection runs, which were distributed all over the aircraft cabin, for example, under the seats, behind the tray tables, in luggage bins, and lavatories. Additionally, six sampling points outside the passenger compartment in the front and rear galley were included (A–C and R–T, Fig. 5). Afterward, two performance runs could be completed using the 180-min incubation protocol with an aPAA concentration of 0.125%. Thereby, the MS2 phage was sensitive to the treatment and reached a >4 log_10_ reduction at 17 out of 20 locations; the carriers at the remaining three locations exhibited a reduction of more than 3 log_10_ levels. For the CSC, a similar result was obtained with 15 out of 20 locations with a reduction >4 log_10_. In the case of the other five locations, a notable reduction could be detected (two below 4 log_10_ and three below 3 log_10_) (Fig. 5; Tables S3 and S4).

**Fig 6:**
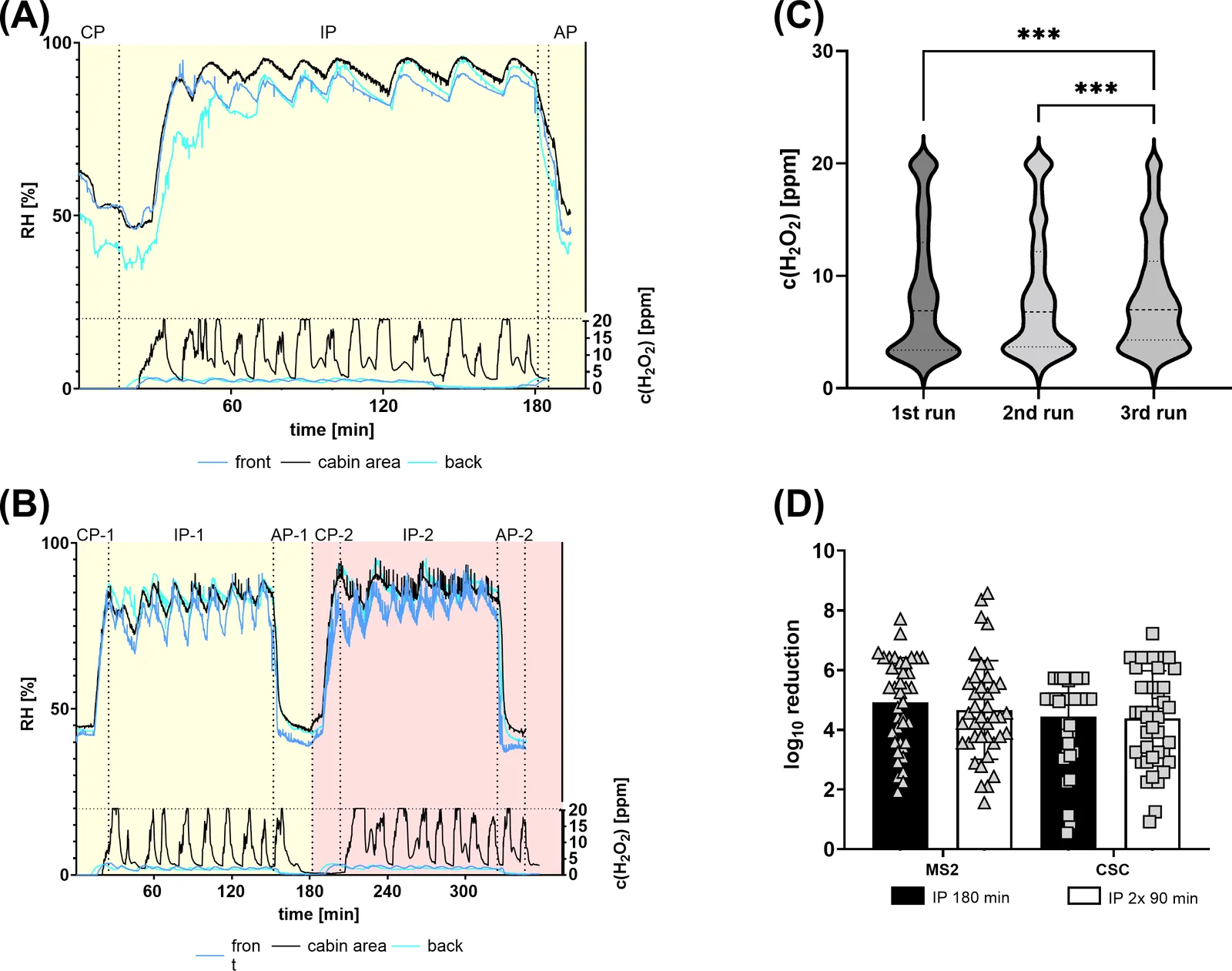
Climate data and HP concentration during disinfection of the aircraft. Representative graphs showing RH and the measured concentration of HP during the disinfection protocol with one incubation phase of 180 min (IP 180 min, **A**) and the disinfection protocol with two incubation phases of 90 min each (IP 2 × 90 min, **B**). (**C**) Comparison of the measured HP concentrations during the three performed runs using the disinfection protocol with two incubation times of 90 min each (IP 2 × 90 min) within the cabin area (upper limit of detection 20 ppm). For all three runs, the values measured at each time point were displayed individually and then compared with each other. A two-way ANOVA following Tukey’s multiple comparisons test was used for statistical comparison. Statistically significant differences are marked with an asterisk: ***: *P*  ≤  0.0005. (**D**) Reduction of MS2 and CSC during IP 180 min (black) and IP 2 × 90 min (white) in the airplane. A two-way ANOVA following Tukey’s multiple comparisons test was used for statistical comparison. Statistically significant differences are marked with an asterisk: ***: *P*  ≤  0.0005.

In order to improve these results, three disinfection runs were performed in the second time slot using the optimized protocol, in which the incubation time of 180 min (IP 180 min) is split into two incubation times of 90 min each (IP 2 × 90 min). Thereby, both incubation phases are separated by an additional aeration and conditioning phase, resulting in a significant increase in the applied amount of aPAA compared to the standard protocol with just one conditioning and a single 180 min incubation phase as shown before (Fig. 2D). Unfortunately, a comparison of the applied amount of PAA during these three disinfection runs revealed an approximate consumption of 80 mL/m^3^ for the first and the second run, but only 73.6 mL/m^3^ for the third one. Due to this deviation of a decisive protocol parameter, the HP concentrations measured at the respective microbial carrier locations over the entire disinfection process of the individual runs were compared with each other. This comparison revealed an overall significantly lower HP concentration during run 3 compared to runs 1 and 2 (Fig. 6C). Since these two key process parameters, the consumption of PAA and the overall distribution of the measurable HP concentration, did not reach the level of the others, the microbiological inactivation results of the third run were not included in the comparative evaluation. Thereby, for the MS2 phage, comparable results were obtained as with the previously used standard protocol (>4 log_10_ reduction at 14 out of the 20 locations; the carriers at the remaining six locations exhibited a reduction of at least >3 log_10_ levels). A similar finding was made for CSC, of which 13 out of 20 locations showed the aimed >4 log_10_ reduction. At the other locations, a strong reduction could be detected, with five being >3 log_10_ and only two below 3 log_10_ levels (Fig. 5 and 6A; Table S4). Furthermore, in contrast to the prior inactivation results in the animal room, no significant difference was observed within the passenger compartment of the aircraft between the two runs with the standard protocol (incubation phase 1 × 180 min) and the two correctly performed runs with the optimized disinfection protocol (incubation phase 2 × 90 min) for both test organisms (Fig. 6D).

During and after the two test periods, the aircraft underwent intensive maintenance and flight approval tests. From a technical perspective, no deviations were detected in the interior of the aircraft. With regard to the general moisture load in the cabin due to the dry fog, no negative impacts were observed either. A before/after measurement of absorbent surfaces such as carpets or leather passenger seat covers revealed only a slight increase of approximately 5%–10% in weight.

## DISCUSSION

Here, we demonstrate that dry fogging of PAA is an effective, standardizable, semi-automatic, and material-friendly room disinfection method suitable for aircraft, which could be used in case of contamination by highly pathogenic bacteria or viruses. Our developed protocol is based on nebulization of aerosolized PAA and can therefore reach otherwise inaccessible areas. Thus, in combination with the standard surface cleaning and wet-disinfection procedures, a safe decontamination of the aircraft could be ensured against both human- and animal-relevant agents. However, the used biocide must be tested under its application conditions with regard to not only its antimicrobial effectiveness but also its material compatibility. For this reason, the aim was to determine the lowest possible yet effective dosage in order to exclude potential effects on the aircraft structure. This differs from previous work in which dry fog was used to decontaminate high-containment rooms or HEPA-filter housings designed for harsh decontamination treatments (23, 24).

At first, a broad range of surrogates covering the different groups of vegetative bacteria, enveloped and non-enveloped viruses, and bacterial endospores were systematically exposed to different concentrations of aPAA to determine the minimal effective concentration for a successful reduction of the specific infectious agents (25). Although the focus was on stainless steel carriers on which the pathogens were air-dried, two types of PVC were also tested to cover relevant material of an aircraft interior. For practical reasons, these initial tests were carried out in a BSC. In order to potentially increase the method’s material compatibility by reducing the minimum effective concentration of aerosolized PAA, the incubation time was increased from 30 to 180 min compared to our previous study (25). Contrary to our expectations, no further reduction in the required minimum concentration of the active ingredient could be determined for the germ carriers with the viral test organisms. In contrast, by extending the exposure time, it was possible to reduce the required minimum active substance concentration for the germ carriers with bacterial spores. This difference is primarily due to the fact that the inactivation results achieved for the various individual runs show significantly smaller differences with this extended exposure time, meaning that the standard error in determining the minimum concentration has a minor influence. However, the overall order of the groups of pathogens in terms of their tolerance to disinfectants (Fig. 1; Table S1), followed the literature (26) and adds to our previous results (25). The obtained data showed that the vegetative bacteria were the most sensitive pathogens, as they could already be sufficiently inactivated at the lowest concentration of aPAA used (0.0078%). Next followed the enveloped viruses, which demonstrated a higher level of variation in their tolerance to aPAA between the selected test organisms (FCoV [0.0078%], MVA [0.0156%], and SARS-CoV-2 [0.0313%]). The larger variability observed between the enveloped viruses tested can be attributed to differences in their respective lipid membrane compositions. For instance, increased resistance to physical and chemical degradation is associated with higher concentrations of aromatic components (27) and saturated fatty acids (28, 29). While poxviruses like MVA are traditionally considered more resilient to chemical inactivation than coronaviruses in liquid suspensions due to their complex, multi-layered membrane architecture, our results demonstrated a slightly higher tolerance of SARS-CoV-2 to aPAA under dried conditions. Beyond differences in lipid membrane composition and the saturation level of fatty acids, this finding can be elucidated by looking at specific structural features: Coronaviruses possess a high density of surface proteins, most notably the heavily N-glycosylated spike (S) protein, which forms a dense carbohydrate and protein “glycan shield” (30). Recent molecular studies have shown that peracetic acid inactivates SARS-CoV-2 specifically by destabilizing and disrupting this spike protein structure and inhibiting its binding to receptors (31). This structural shield may act as a physical barrier that limits or slows down the penetration of aerosolized strong oxidants like peracetic acid to vital viral components, compared to the different protein layout of poxviruses. Furthermore, the experimental setup of the carrier test plays a critical role, as viral susceptibility changes significantly upon drying on surfaces compared to liquid suspensions. During the air-drying process on stainless steel surfaces, the specific cell culture supernatant matrix containing proteins, salts, and cellular debris can induce virus aggregation or micro-encapsulation. Peracetic acid acts as a strong oxidizing agent targeting proteins and nucleic acids; however, if SARS-CoV-2 particles form denser aggregates or are more effectively embedded within a protective organic crust upon drying than MVA, the outermost layers may act as a sacrificial shield against oxidation. Consequently, a marginally higher concentration of the biocide is required to completely penetrate the organic matrix and guarantee full inactivation of the embedded virions (32). However, this does not appear to be universally true for all coronaviruses. FCoV, the standard surrogate test organism for disinfection studies, demonstrated significantly less resilience against aPAA. As expected, the non-enveloped viruses showed an overall stronger tolerance to aPAA, being reduced by >4 log_10_ levels at 0.0313% aPAA only (MNV, ORV-1, MS2, and HAdV-5). The bacterial endospores showed the highest tolerance and could only be inactivated with 0.0625% (*C. difficile*) or 0.125% aPAA (*G. stearothermophilus*). By consistently achieving a reduction of more than 4 log_10_ (99.99%) in highly resistant surrogate organisms, such as the bacteriophage MS2 and *G. stearothermophilus* spores, under realistic aircraft cabin conditions, the remaining level of environmental contamination can be reduced to very low levels. Consequently, the risk of pathogen transmission via contaminated cabin surfaces can be greatly minimized.

The proven microbicidal efficacy observed for the PVC microbial carriers for bacteriophage MS2 could be attributed to the material’s strong hydrophobic surface properties. Since the pathogens were present in either culture medium or other water-based solutions, the spread pathogen suspensions formed more or less a drop on the PVC, resulting in poorer drying results of the pathogens on the carriers and a reduced surface-to-volume ratio and thus a thicker pathogen layer. In contrast to the evenly dispersed pathogen suspensions on the stainless-steel carriers, fewer pathogens were exposed directly to the PAA aerosols. However, this effect was particularly pronounced in the case of the MS2 bacteriophage culture medium, which ultimately could not be distributed on the PVC carrier.

Several pathogens were selected from these data for further testing in the animal facilities to demonstrate the transferability of the protocol to a larger room volume (Fig. S1). As 0.0313% aPAA appeared to be too low for a sufficient reduction of the bacteriophage and insufficient for the bacterial spores in the BSCs, 0.0625% aPAA was set as the minimal concentration for the animal rooms. While the disinfection outcome for the selected viruses and the endospores of *C. difficile* was successful in both animal rooms and could be verified for 0.0625% aPAA, here, the germ carriers with the self-prepared GS could only be sufficiently inactivated with the double concentration (0.125% aPAA), resulting in the same results as the commercial spore carriers of *Geobacillus*. Similarly, for the MS2 bacteriophage, the required aPAA concentration for a >4 log_10_ reduction was observed to be higher in the animal rooms than in the BSC (0.0625% instead of 0.0313%). An analysis of the test parameters showed that the consumption of disinfectant per cubic meter of volume differed from that of the BSC, as more disinfectant was only nebulized at a humidity of less than 85%. Obviously, humidity stabilized better in the animal room than in the BSC, which resulted in a three times lower consumption of PAA (Fig. 2D) and thus in the observed reduced biological effect for GS and MS2 during the experiments. To reduce the possible negative effects resulting from an enrichment of decomposed aPAA during the 180 min incubation time, the protocol for the large room was optimized by splitting it into two incubation phases of 90 min each (IP 2 × 90 min) separated by an additional aeration and conditioning phase (Fig. 2C). Thereby, the lower PAA consumption (mL/m^3^) in the animal room could be significantly increased but was still reduced compared to the amount of that in the BSC (Fig. 2D). This increase in total consumption per m^3^ enabled the necessary inactivation concentration for bacteriophage MS2 with 0.0313% only, while 0.125% aPAA was still required for the commercial microbial carriers with *Geobacillus* spores (Fig. 2E). As the amount of disinfectant aerosols in the room could not be further increased to avoid condensation on surfaces and a wetting of the room, only a higher PAA concentration would increase the biocidal effect. Thus, based on these results, an optimum concentration of 0.125% aPAA would be preferred for the tests in the aircraft.

In general, for the introduction of a new procedure for application on an aircraft, certification in accordance with EASA PART 21/J is required. Thus, the concentration of active ingredient must first pass the official material compatibility tests. To this end, three aPAA concentrations (0.0313%, 0.0612%, and 0.125%) were selected for these crucial tests, and a wide range of relevant and representative aircraft materials (Table 1) were exposed in 10 disinfection runs each. Subsequently, the materials were subjected to a series of material tests in accordance with the currently valid test procedures of aircraft manufacturers as well as aviation standards for the qualification of disinfectants (or similar cleaning and chemical maintenance materials) for use in aircraft. From a technical point of view, these tests were—with a few exceptions—without any anomalies. Only the compulsory flammability tests required for cabin materials (EASA CS25 Appendix F Part I) produced critical results for one seat cover fabric with 0.0313% aPAA. The same was true for a curtain fabric that exhibited a critically extended afterburning time at 0.0625% aPAA. Nevertheless, subsequent retests with higher concentrations or under even more intensive conditions (after direct soaking in the diluted disinfectant) were unable to confirm this. Due to the natural properties of fabric, there may be fluctuations in its flame-extinguishing behavior, which is based on flame retardance. Thus, these tests are the most difficult to perform and often require multiple samples to be tested. Since an increase beyond the permitted limit would mean that these parts are no longer suitable for use in the aircraft in real cases, they have to be replaced with new parts in case of the curtains after a treatment. This procedure will therefore be routinely included in the protocol for a corresponding Lufthansa disinfection process. Before the unclear results for the seat cover fabrics are cleared completely, the disinfection procedure will be limited to aircraft with other seat cover materials. In addition to afterburning tests, corrosion testing of the metals used in aircraft is another crucial procedure. No corrosion occurred when treating all relevant metal alloys with the four different aPAA concentrations used by dry fogging. However, the situation is different when the biocide solution is applied as a liquid film in all concentrations. For this reason, the fogging devices must be filled and emptied outside the aircraft. The third tested decisive material component was two different types of polysulphide sealant material, which is used everywhere on the load-bearing aircraft structure. This proved to be the critical material component in an initial 10-fold treatment using vaporized hydrogen peroxide (VHP) as room disinfection method (33), by which a very strong change in the surface occurred, and a clearly deep-reaching change in the material (>0.5 mm) could be documented in cross-sections (Fig. 4B). As dissolving sealing material is critical for airworthiness and thus not permitted according to “Test Requirement for Sealants in Aircraft” (Ref. D6-7127 [13]) and subsequently the certification specifications CS 25.601 “Design and Construction - General: The aeroplane may not have design features or details that experience has shown to be hazardous or unreliable. The suitability of each questionable design detail and part must be established by tests,” the VHP room disinfection method was ruled out directly after these first test runs. In addition, a possible negative effect of VHP depends on the ambient conditions. As soon as condensation occurs, adverse effects can be observed, such as the hardening of butyl rubber, softening of nylon, or corrosion of metals. Condensation of VHP occurs if there is a difference between the room temperature and the surface temperature, if the humidity rises too much, or if too much VHP is applied to the room. The condensation of VHP can result in a concentration of up to 78% HP in the liquid film formed (34, 35). In contrast, the biocide solution that also contained hydrogen peroxide showed excellent compatibility when aerosolized using the dry fogging method. In this case, only a superficial change was observed at the highest concentration of 1.3%, which resulted in the failure of this MCT, although this did not extend into the depth of the material (Fig. 4C and D). No further change could be detected with the three lower concentrations tested (0.0313%, 0.0625%, and 0.1250%). Thus, after evaluating all of the extensively performed MCTs, PART 21/J Engineering was able to approve the use of a maximum concentration of 0.125% aPAA (with a total duration of 3 h per application) for validation in an Airbus A320. The test suspension was always freshly prepared and used the next day. Initial routine decontamination of affected areas is executed via wet-chemical disinfection utilizing approved antimicrobial agents, contingent upon cabin crew notification regarding infected passengers. Conversely, dry fog decontamination serves exclusively as an ultima ratio measure to be deployed only after all other viable decontamination methodologies have been exhausted. Routine interim cleaning between flight sectors, when conducted at all, cannot be quantified as an effective pathogen eradication measure, given that industry standard practices primarily rely on mild detergents lacking verified disinfectant efficacy. Furthermore, particulate filtration via High-Efficiency Particulate Air (HEPA) systems within the air circulation loop is restricted to specific operational phases of the aircraft. Upon de-energization of the aircraft on the ground, convective transport of pathogen-bearing aerosols occurs unobstructed throughout the entire cabin volume, including all sub-systems engineered for ventilation or decompression. Material compatibility assessments were structured by selecting representative and critical material clusters across both cargo compartments and the passenger cabin, specifically evaluating chemical degradation in elastomers, adhesives, and sealants. Due to the absence of structural obstructions, such as cabin monuments or seating assemblies, within discharged cargo holds, a homogeneous distribution of the specified treatment is readily achievable. While the mandatory sealing of cargo sidewall and ceiling liners for fire-suppressant containment, such as Halon, restricts aerosol propagation into adjacent zones via defined ventilation and decompression pathways, the technical specifications of the deployment equipment have been optimized for volumetric adaptation. Consequently, comprehensive volumetric coverage of the cargo hold geometry is fully ensured, and the structural surfaces of the cargo compartment facilitate efficient wet-chemical disinfection.

Finally, the two different protocols (IP 180 min and IP 2 × 90 min) were tested and compared in an Airbus A320-200 with a cabin size of 165 m³, similar to one of the two animal rooms in which the previous test runs were performed. The upscaling from the BSC to the animal rooms already required an adjustment of the processing parameters and led to different results, so that the adaptation to the airplane was expected to be challenging. Adapting this protocol for a room of the same size but with a different layout and equipment also required modifications to be made. In this case, two dry fogging devices with only one forward-facing and one rear-facing nozzle could be used in order to avoid direct exposure to the overhead luggage bins and the walls of the limited cabin width of narrow-body aircraft such as the A320. This setting ensures that the aerosolized PAA effectively reaches the entire passenger area, resulting in a sufficient inactivation of 4 log_10_ levels (>99.99%) at almost all test locations. However, the front and rear galleys were much less accessible due to the structural partition walls and toilets creating an artificial bottleneck. This is evidenced by the significantly lower relative humidities measured in these areas (Fig. 6A and B). For this reason, both protocols focused on the passenger area for microbiological validation. Additionally, the smooth nature of the surfaces in the galleys enables very effective wet-chemical surface disinfection of these areas. Contrary to expectations based on experiments conducted in the animal room, a direct comparison of microbiological results obtained for two disinfection protocols (IP 180 min and IP 2 × 90 min) revealed comparable microbicidal efficacies, with only minimal differences observed at a few test locations (Fig. 5). Consistent with previous results from tests in the biological safety cabinet and in the animal rooms, the bacterial spores used were more resistant to aPAA than the bacteriophage MS2. Despite the difficulties in reaching the front and rear areas, high inactivation efficiencies of more than 99% were also observed at all tested locations. Nonetheless, according to the collected climate data during all runs, it could be seen that it is challenging to achieve a fully uniform dispersion of the disinfectant aerosols throughout the cabin. Despite the difficulties in achieving a completely uniform distribution of the disinfectant aerosols in the cabin based on the climate data collected during all runs, the inactivation efficiencies achieved here are remarkable. This is particularly impressive, given that the two selected test organisms (bacteriophage MS2 and bacterial spores of *G. stearothermophilus*) are two of the most resistant microorganisms against aPAA, as demonstrated here. As most relevant pathogens with zoonotic potential are enveloped viruses with a significantly higher sensitivity to aPAA, this method should be even more effective in these cases. Nonetheless, the lack of difference in microbiological efficacy between the two protocols is due to the multiple hidden cavities and multiple compartments in the aircraft and the corresponding tiny connections between them. These conditions were thus comparable to those in the BSC, which was used for the initial tests and in which the installed HEPA filters continuously absorbed part of the aerosolized disinfectant solution throughout the entire incubation period. For this reason, newly generated PAA aerosols had to be regularly added during the incubation period in both the BSC and the aircraft (Fig. 2A for the BSC and Fig. 6A and B for the aircraft) to maintain the desired relative humidity of approximately 90%. Due to the regular addition of fresh disinfectant, it could not be proven that exchanging the entire amount of disinfectant applied in the optimized protocol (IP 2 × 90 min) increased the inactivation efficiency, as it was the case for the hermetically sealed animal room in which only minimal fresh disinfectant had to be added during the incubation phase (Fig. 2B and C). Another important factor observed for the disinfection efficacy was the climate conditions on the particular day and the initial humidity. On very humid days, we found that with an initial relative humidity of more than 60%, not enough PAA aerosols, which mainly comprise water, could be fumigated into the rooms until saturation was reached, whether in a safety cabinet or an animal facility. The application of our aerosol-based disinfection would thus be limited to a climate condition below 60% RH (36). Additionally, to minimize disruptive climatic effects during the preconditioning phase and maintain the necessary climatic parameters inside the aircraft cabin, the aircraft was parked in a hangar during both entire validation periods. However, the optimized protocol (IP 2 × 90 min) should demonstrate higher microbiological efficacy at an initial relative humidity of over 55%, due to the one-time exchange of the entire applied disinfectant and the significantly higher amount of disinfectant applied, which is comparable to the results obtained in the animal room. Therefore, the optimized protocol is significantly more flexible and robust than the protocol with a single incubation phase (IP 1 × 180 min) and thus the procedure of choice. Eventually, it should be noted that good ventilation is crucial after the end of the process in order to quickly and effectively remove the applied mixture of active substances from the aircraft. Further tests should still be conducted at this stage of the project to determine whether the applied biocide mixture can also be used to actively circulate for treating and decontaminating the airpipes and the installed HEPA filters by starting the aircraft’s ventilation system in advance (36). Although there is a connection between the passenger and the cargo compartment, we could not detect any HP as a marker of biocide aerosols within the cargo compartment in the current experiments. This would further reduce the risk of exposure to personnel or the environment during the mandatory filter change specified for a contamination case, which is carried out using a bag-in-bag process. To effectively ventilate the aircraft afterward, all doors should be opened, and large fans should be placed in the front or rear galley area. The ventilation period should also be sufficient for at least 24 h to allow a complete outgassing and ventilation of aPAA and degradation products that have accumulated in the carpet and in foam parts behind inaccessible fixtures; otherwise, the detectable disinfectant concentration would always equalize within the cabin space due to diffusion processes. Concerning the technical point of view, it was shown that the dry fog technology had no negative impact on the aircraft cabin, the interior, and existing systems in the application specification described. All system and functional tests carried out immediately afterward were completed without any findings. Furthermore, none of the validation runs in the aircraft revealed any residual moisture ingress. A corresponding “Engineering Bulletin Simplified Aircraft/Engine Change (EB-SC)” was finalized describing the application of the optimal protocol (IP 2 × 09 min) using 0.125% aPAA in narrow-body aircraft (File S2). This breakthrough now enables the first-ever effective, material-compatible process for disinfecting aircraft cabins, which can be adapted to other aircraft models with minimal effort. However, the frequency of use per aircraft still should be limited and thus only be carried out in the context of an incidence with a high-consequence infectious disease on board to keep the impact of the procedure to a minimum.

## MATERIALS AND METHODS

### Viruses and cells

Relevant representatives of viruses in human and veterinary medicine were selected (Table 2), which cover the different classes such as enveloped and non-enveloped viruses, as well as DNA and RNA viruses.

**TABLE 2:** Summary of viruses and cells^*a*^

Virus	Properties	Cell line/host cell	Medium
HAdV-5 TCID_50_/mL: 7.58 × 10^6^ Source: ATCC, VR-1516	dsDNA, non-enveloped	A549 lung (*Homo sapiens*) Source: DSZM GmbH, ACC 107	MEM, HEPES, NEA, sodium pyruvate, P/S, L-glutamine
FCoV, strain “Munich” TCID_50_/mL: 2.95 × 10^7^ Source: RVB, RVB-1259	ssRNA, enveloped	CRFK kidney of cat (*Felis catus*) Source: RZB, CCLV-RIE 0115	MEM (E), MEM (HBSS), NaHCO_3_, sodium pyruvate, NEA
MNV, strain “S99” TCID_50_/mL: 1.78 × 10^9^ Source: RVB, RVB-0651	ssRNA, non-enveloped	RAW 264.7 Ascites of mouse strain BALB/c (*Mus musculus*) Source: RZB, CCLV-RIE 0996	MEM (E), MEM (HBSS), NaHCO_3_, sodium pyruvate, NEA
MS2 TCID_50_/mL: 2.95 × 10^8^ Source: DSZM GmbH, DSM number: 13767	ssRNA, non-enveloped	*E. coli* Lederberg W1485 Source: DSZM GmbH, 13767	LB medium
MVA TCID_50_/mL: 1.05 × 10^7^ Source: Ludwig-Maximilians-Universität München	dsDNA, enveloped	BHK-21 kidney of hamster (*Mesocricetus auratus*) Source: DSZM GmbH, ACC 61	MEM (E), MEM (HBSS), NaHCO_3_, sodium pyruvate, NEA
ORV-1, strain “Erik” TCID_50_/mL: 4.90 × 10^7^ Source: RVB, RVB-0391	dsRNA, non-enveloped	ST testis of domestic pig (*Sus scrifa domestica*) Source: RZB, CCLV-RIE 0606	DMEM, P/S, L-glutamine
SARS-CoV-2, strain “BavPat1/2020” TCID_50_/mL: 4.27 × 10^6^ Source: Bundeswehr Institute of Microbiology	ssRNA, enveloped	Vero E6 kidney of African green monkey (*Chlorocebus sabaeus*) Source: RZB, CCLV-RIE 0929	MEM (E), MEM (HBSS), NaHCO_3_, sodium pyruvate, NEA

^*a*^ HAdV-5, Human mastadenovirus C; FCoV, Feline coronavirus; MNV, Murine norovirus 1 strain; MS2, Emesvirus zinderi (Escherichia phage); MVA, Modified Vaccinia Ankara; ORV-1, Mammalian orthoreovirus 1; SARS-CoV-2, severe acute respiratory syndrome coronavirus 2; ATCC, The Global Bioresource Center; RVB, Riemser Virusbank, FLI, Germany; RZB, Riemser Zellbank, FLI, Germany; DSMZ, German Collection of Microorganisms and Cell Culture GmbH; NEA, non-essential amino acids; MEM (E), minimum essential medium with Earle’s salts; MEM (HBSS), minimum essential medium with Hanks' balanced salts; P/S, penicillin/streptomycin.

The virus stocks were prepared with titers of at least 4 × 10⁶ TCID_50_/mL so that a reduction of at least 4 log levels could be demonstrated. Therefore, monolayer cell cultures in T175 flasks were inoculated with 1 mL of the virus in a final volume of 6 mL medium (without FCS) at 37.0°C, 5% CO_2_, 95.0% RH. After 1 h, the medium (with 2% FCS) was added, and the cells were incubated for 1–3 days. For harvest, the flasks were frozen at −80°C and thawed; the lysed cells were collected and centrifuged at 340 *× g* for 10 min. The supernatant was aliquoted and stored at −80°C until use. Virus titers were determined by the median tissue culture infectious dose (TCID_50_) assay by counting the wells of 10-fold serial dilutions showing cytopathic effect after 3–7 days of incubation depending on the virus. The TCID_50_/mL values were calculated using the method described by Spearman and Kärber (37, 38). The prepared viruses were aliquoted and stored at −80°C until use.

### Propagation of *Escherichia* phage MS2 (*Emesvirus zinderi*)

*Escherichia coli* was used for propagation of the *Escherichia* phage MS2. Equal volumes of *E. coli* and the bacteriophage were mixed in a reaction tube and incubated for 10 min at room temperature. This mixture was added to soft agar (LB with 0.7% agar agar) and poured over a solid LB agar plate (1.5% agar agar). The plate was incubated overnight at 37°C and then transferred into SM buffer (5.8 g/L NaCl, 2.0 g/L Mg_2_SO_4_ × 7 H_2_O, 5% [vol/vol] 1 M Tris-HCl). After stirring for at least 4 h, the suspension was centrifuged at 3,200 *× g* for 30 min. The supernatant was filtered (0.45 µm < followed by 0.2 µm) and stored in a refrigerator. To quantify the bacteriophage, it was titrated in LB medium on a 96-well plate. *E. coli* was mixed in soft agar and poured onto a solid 12 × 12 cm LB agar plate. Each dilution step of the 96-well plate was dropped (10 µL/drop) onto the surface of the agar plate and evaluated the next day for the appearance of plaques. Concentration was calculated using the TCID_50_ method (37, 38).

### Cultivation of bacteria

*Acinetobacter baumannii* (DSMZ 30007) was cultivated on tryptone-soy agar for 24 h at 36°C before harvest. After filtration over glass wool, the pellet was washed twice prior to suspending in ELGA Ultra water by vortexing for 1 min with glass balls to obtain the test suspension. The titer was adjusted to 1 × 10^8^ CFU/mL. The test suspension was always freshly prepared and used the next day. Quantification was carried out by plate count on the next day after incubation at 36°C.

*E. hirae* (ATCC 10541) and *S. aureus* (ATCC 6538) were freshly cultivated as overnight cultures for 20 h at 37°C and 200 rpm in tryptone-soy broth or Müller-Hinton broth, respectively. The titer was adjusted to approximately 1 × 10^8^ CFU/mL by optical density determination. The test suspension was always freshly prepared and used the next day. Quantification of the suspensions was carried out by plate count after overnight incubation at 37°C.

### Preparation of endospores

*C. difficile* (NCTC 13366, DSMZ 27147) spore suspension was prepared, and the quality was confirmed according to VAH guidelines (method 18; https://vah-online.de/files/download/ebooks/eBook_VAH_Methoden_Anforderungen.pdf). The working stock was diluted to 1 × 10^8^ CFU/mL in ELGA Ultra water. Quantification was carried out by plate count on brain-heart-infusion agar (Oxoid, UK; supplemented with 0.5% yeast, 0.1% L-cysteine, and 0.1% taurocholate) under anaerobic conditions for 48 h at 36°C.

Commercial *Geobacillus stearothermophilus* spores (CSC) were purchased as Apex Ribbons (Mesa Labs, France) coated with 1.7 × 10^6^ *G. stearothermophilus* spores (ATCC 12980) with an indicated thermal resistance (D value) of 2.7 min in 2 mg/L gaseous hydrogen peroxide as well as a suspension with 1.2 × 10^8^ *G. stearothermophilus* spores per mL (ATCC 7953; Liofilchem, Italy) with a D value of 1.5 min in gaseous hydrogen peroxide were used as standardized surrogate in all experiments at the different sites. Spores were cultivated in tryptone-soy broth for 48 h at 60°C and quantified by estimating the most probable number (39).

### Germ carrier preparation and analysis

Germ carriers were prepared as described elsewhere (25). Briefly, commercial spore carriers (Mesa Labs, France) coated with *G. stearothermophilus* spores, as well as self-prepared germ carriers, were used. Stainless steel (GK Formblech, Germany), a polyvinyl chloride reference (GK Formblech, Germany), and polyvinyl chloride aircraft seat material (provided by Lufthansa Technik AG) were used to prepare carriers measuring 16 by 60 mm. These carriers were coated with 50 µL of the respective microorganism and evenly spread. After air drying in a working BSC, germ carriers were exposed to the disinfectant during the experiments. For the analysis of the remaining infectivity, microorganisms were collected in 1 mL of the respective medium by rinsing the germ carrier 10 times and subsequently titrated. Depending on the microorganism, plate count or TCID_50_/mL was performed for titer determination. A disinfection procedure is successful if it reduces >5 log_10_ units of bacteria or >4 log_10_ units of viruses and bacterial spores, respectively.

### Material compatibility testing

Based on the preliminary tests carried out, a comprehensive test set was compiled from various relevant aircraft materials. This test set includes the materials listed in Table 1. The prepared material samples were placed in a room intended for animal husbandry at FLI, in which the biological validation was performed, too. The fabric samples and the insulating mats were hung in the room (Fig. 3).

### Methods relevant for airworthiness with safety aspects

#### Sandwich corrosion test

The Sandwich corrosion test (ASTM-F1110 [21]) defines the procedure for evaluating the corrosivity of aircraft maintenance chemicals, when present between faying surfaces (sandwich) of aluminum alloys commonly used for aircraft structures.

During the test, two filter paper strips soaked with the active ingredient are placed between two identical aluminum sheet samples (so-called sandwich structure) and exposed to a climate cycle for 168 h. After completion of the climatization, which can be compared with artificial aging in fast motion, the samples are evaluated regarding possible corrosion and material changes.

In addition to the above-specified procedure, LHT Engineering elaborated alterations to better simulate the actual impact of the applied dry fog on the sandwich structure. As a result, three sample sets placed inside the test room were treated differently between the 10 test cycles as follows:

- 1× open, afterward 9× sealed closed (sandwich),
- 10× open, afterward sealed closed (sandwich), and
- 10× open, 10× filter paper soaked in demineralized water in-between dry fog application, afterward sealed closed (sandwich). Immediately after the last of the 10 dry fogging cycles, the samples were individually packed airtight in sealed bags and thus preserved until the start of climatic conditioning in the test laboratory.

#### Flammability properties

The change in the flame-retardant property is tested by means of a flammability test [Bunsen burner test, EASA CS 25 Appendix F, Part I (a) (40)] on differently contaminated and uncontaminated fabric, plastic, and carpet samples, as well as laminates. The possible change in heat release was also determined in a heat release rate test in accordance with EASA CS 25 Appendix F, Part IV by exposure to disinfectants on different materials in comparison with uncontaminated materials. A test in accordance with EASA CS 25 Appendix F, Part VI (40) was carried out specifically for thermal/acoustic insulation materials to assess flame propagation and flammability.

Furthermore, the possible influence on the smoke emission of large-area cabin components (e.g., decorative foils) was checked using a smoke density test. (EASA CS 25 Appendix F, Part V [40]).

#### Material changes in elastomers, tapes, and sealants

The influence of disinfectants on the strength and elasticity of elastomers is tested using a tensile test in accordance with Boeing D6-7127 (13) specifications. Possible swelling or surface attack on the elastomer is also tested and subsequently assessed as “Pass” or “Fail” (Boeing D6-7127 [13]). This applies to sealants and silicone as a substitute for other elastomers used in the aircraft. Further material damage, such as changes in adhesion and color of sealants and primers, is investigated and evaluated (Boeing D6-17487 [14]).

#### Polymer stress crazing

In the stress crazing test (ASTM-F484 [41]), acrylic glass is tested to determine whether the disinfectant triggers stress cracks. According to the standard, the test strips were kept under constant tension in a device in the test room for the entire time of the dry fogging, for example, 10 times, and after the test, the area of the highest stress was examined and evaluated regarding crack development.

#### Solubility in condensed water

Approximately 250 mL of demineralized water was exposed to 10 dry fogging applications in an open container in the test room to simulate whether one of the disinfectants, hydrogen peroxide or peracetic acid, concentrates in the condensed water that forms in an aircraft cabin during normal operation. The concentration would result in a high risk of corrosion.

After the tenth dry fogging cycle, the concentrations of hydrogen peroxide and peracetic acid in water were determined in the test room using semi-quantitative test strips (Macherey-Nagel Quantofix Peroxide 25, Peroxide 1000, and Peracetic acid 500).

#### Paint-softening test

In a deviation from the specification ASTM F502 (18), various plastic and painted surfaces were tested for possible paint-softening effects of disinfectants, which can lead to damage by impairing the corrosion protection of components. A comparison of the scratch resistance of the surface to ascending pencil hardness used in the ASTM F502 (18) standard was carried out. As a rule, the test is considered failed if a difference of two pencil hardnesses is determined between the loaded and unloaded reference surface.

### Methods for assessing surface changes without a safety aspect

#### Test for signs of aging

Possible visible signs of aging caused by disinfectants were tested for leather, artificial leather, carpet, synthetic floor coverings, and various textile materials (DIN EN ISO 105-A03:2020-02 [42] and DIN EN 13018:2016-02 [43]). Material surfaces were checked by optical comparison with uncontaminated materials using a gray scale for evaluating the change in color.

### Nebulization of peroxyacetic acid under lab conditions

For the laboratory experiments, ultrafine dry fog was generated in a biological safety cabinet (internal volume 1 m³) using a Mini Dry Fog system or in an animal room (21 m³ or 164 m³) using a Dry Fog 2-S system (IPM Filtration Systems, Italy) (25, 44). Compressed air was generated with a mobile air compressor (model C330/03 Gentilin, Italy). Working solutions of the disinfectant were obtained by mixing stock solutions of 4.84% (vol/vol) PAA (Lerasept Spezial, Stockmeier Chemie GmbH & Co. KG, Germany) with deionized water. All concentrations used and indicated refer to the PAA contained in the solution. The concentration of the hydrogen peroxide also contained in the solution is approximately five times higher.

In case of the treatment of the whole room, radio-controlled sockets were used to remotely operate the compressor from outside of the room. For both set-ups, the biosafety cabinets and the rooms were sealed using tape, and ventilation was stopped (BSC was turned off). During the course of nebulization, the relative humidity and temperature were recorded using data loggers (model 635-2; Testo, Germany). Experiments were started at 40%–60% RH. The disinfectant was nebulized until the RH reached 90%–95% as a conditioning of the room and was then frequently applied to keep a constant level between 85% and 95% RH. After incubation for 180 min, an aeration phase of 30 min followed. In a final experiment, the impact of an intermediate ventilation cycle was examined. For this, the incubation phase of 180 min was divided into 2 × 90 min with an additional aeration phase of 30 min in between to remove the dry fog before starting the second cycle.

#### Nebulization of aPAA in a passenger aircraft

To test and validate the dry fog procedure in an airworthy aircraft, an Airbus A320-200 was provided by LHT at two distinct time slots. During the whole period, the aircraft was parked in a hangar to minimize climatic variations. Two Dry Fog 2-S machines with two nozzles each in the longitudinal direction were set up in the cabin (Fig. 5). For an unhindered airflow and thus aerosol dispersion in the cabin, all luggage bins were opened, tray tables were set down, and trolleys were removed, as well as one door of the rear lavatories was dismounted, and the other was fixed in an open position. The cockpit door to the cabin was sealed, and all doors were closed tightly except the front left door, which was left ajar and sealed with tape to enable the routing of the power cables of the compressors. The compressors were remotely operated by using radio remote-controlled sockets from outside of the aircraft. In the course of nebulization, temperature and RH were recorded using data loggers at each of the 20 germ carrier locations (14 in the passenger area and additionally 6 in the front and back parts of the aircraft, Fig. 5, 6A and B; Fig. S2). In addition, the concentration of hydrogen peroxide was documented at three selected locations (Fig. 5, 6A and B), as this is the only component of the PAA mixture that can be reliably measured in the air using a sensor. First, the airplane was conditioned with dry fog to 85%–90% RH before the incubation phase (180 or 2 × 90 min) started with frequent administration of the aerosols to keep a stable humidity. In case of the protocol with the divided incubation phase, an additional aeration phase of 30 min using four fans (Heylo PowerVent 6000) placed in the front area of the aircraft and all doors opened was applied before the second disinfection cycle was started and performed in the same way, with the conditioning of the cabin first, followed by the second incubation phase of 90 min. Subsequently, within the final aeration, the cabin was ventilated by opening all four doors of the aircraft and using four fans placed in the front area until hydrogen peroxide content reached 0.0 ppm at all three locations. Afterward, the germ carriers were collected for microbiological processing.

## SUPPLEMENTAL MATERIAL

File S1 msphere.00542-26-s0001.pdf Fig. S1 and S2; Tables S1 to S4.

File S2 msphere.00542-26-s0002.pdf Generated final “Engineering Bulletin Simplified Aircraft/Engine Change” for application option.

ASM does not own the copyrights to Supplemental Material that may be linked to, or accessed through, an article. The authors have granted ASM a non-exclusive, world-wide license to publish the Supplemental Material files. Please contact the corresponding author directly for reuse.
