# Supplementary material for: Aerosol-based disinfection protocol for aircraft: a solution for the conflict between material compatibility and antimicrobial efficacy
Source: mSphere. 2026 Aug 31;11(9):e00542-26. doi: 10.1128/msphere.00542-26 (PMC13639886; doi:10.1128/msphere.00542-26)
Supplement: Supplementary file 1 [file msphere.00542-26-s0001.pdf]

Supplements

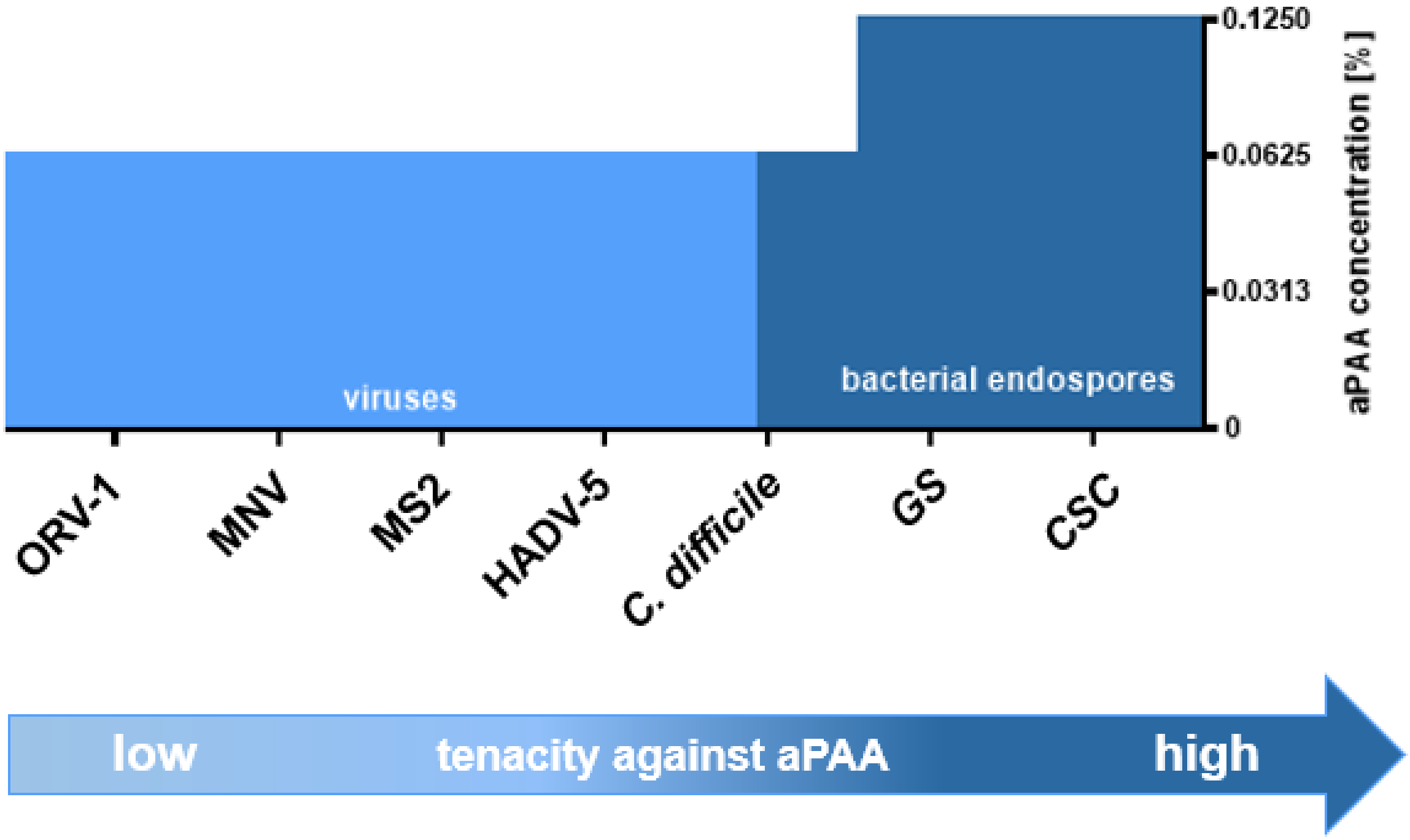

Figure S1. **Confirmation of the effective concentration of aerosolized peracetic acid (aPAA; 0.0625% and 0.125%) for enveloped and non-enveloped viruses and bacterial spores.** The effective concentrations of aPAA is shown that was necessary to reach the required reduction (viruses and bacterial endospores > $4\log_{10}$ levels and vegetative bacteria > $5\log_{10}$ levels; stainless steel germ carriers were inoculated with MVA, Modified-Vaccinia-Ankara-Virus; MNV, murine norovirus; ORV-1, Mammalian orthoreovirus 1; MS2, Escherichia phage MS2; HAdV-5, Human adenovirus 5; C. difficile, *Clostridioides difficile* endospores; GS, *Geobacillus stearothermophilus* endospores; CSC, commercial spore carriers coated with *GS* endospores; n ≥ 3).

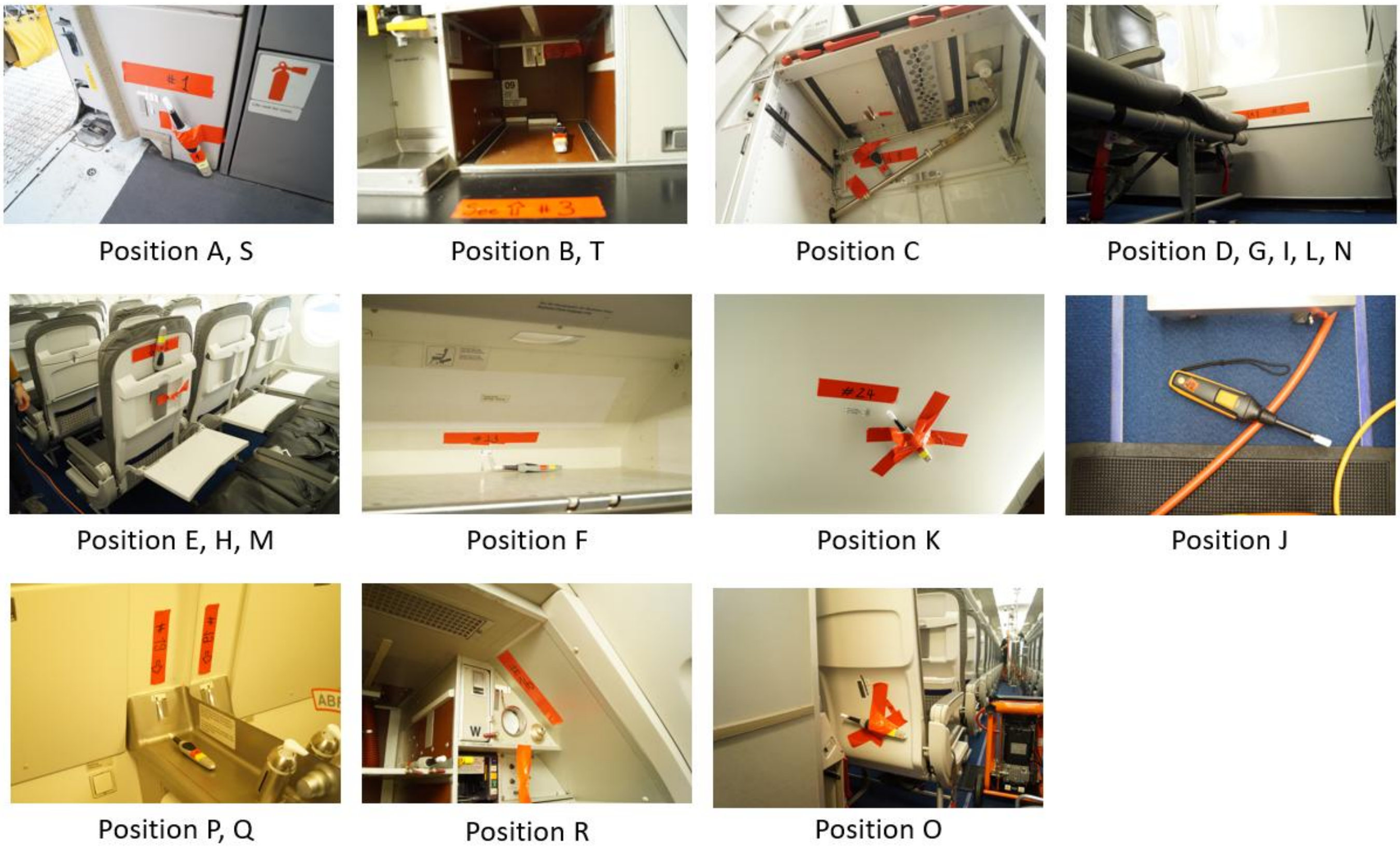

Figure S2. **Locations of the germ carriers in the aircraft.** D. G. I. L. N under a seat; positions: E. H. M. O backrest; positions: A. R. S aircraft door; positions: B. C. T galley; positions: P. Q lavatories; position: F luggage bin; position: K ceiling; position: J floor

Table S1. Overview of the tenacity of microorganisms against aerosolized peracetic acid (aPAA) when dried on stainless steel, PVC Kömadur ES and polyvinyl chloride aircraft seat material and exposed for 180 min. Microbicidal safety cabinet (MSC).

microorganism	c (aPAA) in %	stainless steel	PVC Kömadur ES	PVC aircraft seat material
A. baumannii	0.0078	5.71±0.61	n.d.	n.d.
	0.0156	5.71±0.22	n.d.	n.d.
	0.0313	5.79±0.43	n.d.	n.d.
E. hirae	0.0078	6.47±0.11	n.d.	n.d.
	0.0156	6.45±0.02	n.d.	n.d.
	0.0313	6.25±0.04	n.d.	n.d.
	0.0625	5.77±0.53	n.d.	n.d.
S. aureus	0.0078	5.93±0.07	n.d.	n.d.
	0.0156	6.02±0.09	n.d.	n.d.
	0.0313	6.32±0.05	n.d.	n.d.
	0.0625	5.90±0.05	n.d.	n.d.
FCoV	0.0078	4.80±0.42	n.d.	n.d.
	0.0156	4.63±0.21	4.73±0.15	4.46±0.17
	0.0313	5.03*±0.36	5.09±0.13	4.94±0.17
	0.0625	6.14*±0.91	4.48±0.18	4.73±0.30
MVA	0.0078	2.25±0.23	n.d.	n.d.
	0.0156	5.05±0.15	5.51±0.17	n.d.
	0.0313	5.24±0.13	5.59±0.50	n.d.
	0.0625	5.85±0.11	5.25±0.21	n.d.
MNV	0.0078	1.19±0.65	n.d.	n.d.
	0.0156	4.36±0.41	4.44±0.25	4.67±0.28
	0.0313	4.60±0.12	5.51±0.31	5.95±0.22
	0.0625	5.75*±0.14	5.91±0.24	5.05±0.14
	0.1250	n.d.	5.26±0.53	4.84±0.41
ORV-1	0.0078	0.34±0.41	n.d.	n.d.
	0.0156	1.91±0.32	2.50±1.17	n.d.
	0.0313	5.79*±0.11	5.24±0.45	n.d.
	0.0625	5.00*±0.14	4.86±0.50	n.d.
SARS CoV-2	0.0078	3.38±0.44	n.d.	n.d.
	0.0156	4.00±0.45	n.d.	n.d.
	0.0313	5.17±0.13	n.d.	n.d.
MS2	0.0078	0.64±0.17	n.d.	n.d.
	0.0156	2.93±0.57	1.02±0.12	1.41±0.24
	0.0313	4.57±0.18	2.66±0.13	4.65±0.50
	0.0625	6.58±0.74	3.97±0.15	4.43±0.37
	0.1250	6.10±0.31	4.51±0.34	5.22±0.21
HAdV-5	0.0078	0.35±0.25	n.d.	n.d.
	0.0156	3.24±0.43	2.67±0.48	n.d.
	0.0313	4.63±0.26	3.96±0.40	n.d.
	0.0625	5.40±0.12	5.14±0.26	n.d.
C. difficile	0.0313	2.07±0.26	0.62±0.09	n.d.
	0.0625	7.15±1.12	4.68±1.62	n.d.
GS	0.0156	1.21±0.46	2.72±0.98	2.06±1.20
	0.0313	3.31±0.80	6.60±0.36	5.53±0.27

	0.0625	5.74±0.25	5.63±0.20	6.13±0.22
	0.1250	5.44±0.48	6.24±0.50	5.72±0.26
CSC	0.0078	-0.28±0.07	n.d.	n.d.
	0.0156	0.24±0.11	n.d.	n.d.
	0.0313	0.44±0.27	n.d.	n.d.
	0.0625	3.20±0.27	n.d.	n.d.
	0.1250	5.19±0.32	n.d.	n.d.

Reduction in Log_{10} $TCID_{50}$/ml (weighted average ± consistency); c(aPAA). concentration of aerosolized peroxyacetic acid; *A. baumannii. Acinetobacter baumannii; E. hirae. Enterococcus hirae*; *S. aureus. Staphylococcus aureus*; FCoV. Feline coronavirus; MVA. Modified-Vaccinia-Ankara-Virus; MNV. murine norovirus; ORV-1. Mammalian orthoreovirus 1; SARS-CoV-2. severe acute respiratory syndrome coronavirus 2; MS2. Escherichia phage MS2; HAdV-5. Human adenovirus 5; *C. difficile. Clostridioides difficile* endospores; GS. *Geobacillus stearothermophilus endospores*; CSC. commercial spore carriers coated with GS endospores; * complete reduction; n.d. not determined

Table S2. Overview of the tenacity of microorganisms against aerosolized peracetic acid (aPAA) when dried on stainless steel and exposed for 180 min or 2x 90 min, respectively, in an animal room.

microorganism	c (aPAA) in %	animal room (IP = 180 min)	animal room (IP = 2x 90 min)
MNV	0.0625	5.61±0.12	n.d.
	0.125	4.52±0.29	n.d.
ORV-1	0.0625	4.75±0.05	n.d.
MS 2	0.0313	2.14±0.41	5.13±0.43
	0.0625	4.57±0.24	n.d.
	0.1250	6.57±2.10	6.30±0.20
HADV-5	0.0625	5.93±0.13	n.d.
C. *difficile*	0.0625	6.69±0.10	n.d.
GS	0.0625	3.41±0.24	n.d.
	0.125	6.69±0.10	n.d.
CSC	0.0078	n.d.	n.d.
	0.0156	n.d.	n.d.
	0.0313	n.d.	n.d.
	0.0625	3.94±0.33	n.d.
	0.1250	6.49±0.32	5.88±0.10

Reduction in Log_{10} $TCID_{50}$/ml (weighted average ± consistency); c(aPAA). concentration of aerosolized peroxyacetic acid; MNV. murine norovirus; ORV-1. Mammalian orthoreovirus 1; MS2. Escherichia phage MS2; HADV-5. Human adenovirus 5; C. *difficile*. *Clostridioides difficile* endospores. GS. *Geobacillus stearothermophilus endospores.* CSC. commercial spore carriers coated with GS endospores; n.d. not determined

Table S3. Reduction of CSC and MS2 in the aircraft (IP 180 min. c(aPAA)=0.125%)

position	1st run (CSC)	2nd run (CSC)	mean of 1st and 2nd run (CSC)	1st run (MS2)	2nd run (MS2)	mean of 1st and 2nd run (MS2)
A	5.73	2.33	4.03	6.42	6.25	6.34
B	2.24	1.14	1.69	2.42	5.25	3.84
C	3.24	4.21	3.73	5.42	4.25	4.84
D	3.86	5.03	4.45	6.42	3.92	5.17
E	5.73	5.03	5.38	6.42	6.58	6.50
F	5.73	5.03	5.38	5.42	4.92	5.17
G	0.88	4.15	2.52	4.42	5.92	5.17
H	0.54	5.03	2.79	5.42	5.58	5.50
I	3.83	5.03	4.43	7.22	7.72	7.47
J	3.24	5.03	4.14	4.42	3.58	4.00
K	5.73	5.03	5.38	4.75	5.92	5.34
L	3.54	3.13	3.34	3.42	4.25	3.84
M	3.03	5.03	4.03	4.08	6.25	5.17
N	5.65	5.03	5.34	3.08	2.92	3.00
O	5.73	5.03	5.38	6.42	3.58	5.00
P	5.73	5.03	5.38	6.08	3.58	4.83
Q	5.73	5.03	5.38	6.08	3.25	4.66
R	5.73	5.03	5.38	6.42	1.92	4.17
S	5.73	5.03	5.38	6.42	2.58	4.50
T	5.73	4.95	5.34	5.42	2.25	3.84

Reduction in Log_{10} $TCID_{50}$/ml (weighted average ± consistency); c(aPAA). concentration of aerosolized peroxyacetic acid; CSC. commercial spore carriers coated with GS endospores; MS2. Escherichia phage MS2

Table S4. Reduction of CSC and MS2 in the aircraft (IP 2x 90 min. c(aPAA)=0.125%)

position	1st run (CSC)	2nd run (CSC)	3rd run* (CSC)	mean of 1st and 2nd run (CSC)	1st run (MS2)	2nd run (MS2)	3rd run* (MS2)	mean of 1st and 2nd run (MS2)
A	2.92	6.42	6.25	4.67	5.56	4.44	4.33	5.00
B	4.58	2.42	5.25	3.50	3.56	3.78	1.67	3.67
C	3.25	5.42	4.25	4.34	1.56	2.78	3.00	2.17
D	4.58	6.42	3.92	5.50	6.22	3.78	3.33	5.00
E	5.25	6.42	6.58	5.84	6.22	7.78	0.67	7.00
F	3.92	5.42	4.92	4.67	6.56	5.78	3.33	6.17
G	1.25	4.42	5.92	2.84	4.22	4.44	3.00	4.33
H	4.92	5.42	5.58	5.17	4.22	4.78	3.67	4.50
I	6.05	7.22	7.72	6.64	8.36	8.58	5.33	8.47
J	2.25	4.42	3.58	3.34	5.22	5.78	5.00	5.50
K	3.25	4.75	5.92	4.00	7.56	5.44	5.33	6.50
L	2.25	3.42	4.25	2.84	4.56	4.78	3.33	4.67
M	2.92	4.08	6.25	3.50	3.56	2.44	2.67	3.00
N	3.58	3.08	2.92	3.33	3.89	2.11	2.67	3.00
O	4.58	6.42	3.58	5.50	5.56	3.78	4.33	4.67
P	3.92	6.08	3.58	5.00	4.22	4.44	3.00	4.33
Q	3.25	6.08	3.25	4.67	5.22	3.11	2.67	4.17
R	0.92	6.42	1.92	3.67	2.89	3.78	2.67	3.34
S	2.58	6.42	2.58	4.50	3.56	5.44	4.00	4.50
T	2.92	5.42	2.25	4.17	4.56	2.11	1.67	3.34

Reduction in Log_{10} $TCID_{50}$/ml (weighted average ± consistency); c(aPAA). concentration of aerosolized peroxyacetic acid; CSC. commercial spore carriers coated with GS endospores; MS2. Escherichia phage MS2; *Data from the third run not analysed due to the low hydrogen peroxide concentration
