# Supplementary material for: Aerosol-based disinfection protocol for aircraft: a solution for the conflict between material compatibility and antimicrobial efficacy
Source: mSphere. 2026 Aug 31;11(9):e00542-26. doi: 10.1128/msphere.00542-26 (PMC13639886; doi:10.1128/msphere.00542-26)
Supplement: Supplementary file 2 [file msphere.00542-26-s0002.pdf]

# Engineering Bulletin Simplified Aircraft/Engine Change (EB-SC)

**Lufthansa Technik**
**Design Organisation**

## EB-SC-No. DES_119502/1

Project-No. 00173859

**Title**
Disinfection of Cabin Interior using Dry Fogging with Lerasept® Spezial

| **Product / (A/C type or Engine type)** | **ATA** | **OEM-Reference** (e.g. SB, AMM ,EM ,CMM, IPC) |
|---|---|---|
| A320/ ALL | 25-00-00 | AMM |

| **Part Denomination** | **PNR** | **SNR** | **Part Manufacturer** |
|---|---|---|---|
| Lerasept® Spezial | | | Stockmeier Chemie GmbH & Co.KG |

| **Restriction** | | | **Aircraft/Engine Status** | | |
|---|---|---|---|---|---|
| MSN/ESN | Tailsigns | Operator/Customer | Hours | Cycles | Date |
| | D-AOJT | LHT | | | |

**Reason for Revision**
Initial Revision

**Brief Description of Change**
Part of Change which is covered by already approved Data (Manufacturer Approval / Manual)
Pre-conditioning of cabin environment using a low pressure ground connection per AMM TASK 12-33-21-618-001-A03 or through the HP Ground Connection i.a.w AMM TASK 12-33-21-618-001-A01.
Part of Change for which LHT-DO takes the responsibility and provides the showing of compliance
Disinfection of Cabin Interior using dry fogging procedure with disinfectant Lerasept® at a max. concentration of 0,125% PAA.

**Affected Documents and Data** ( e.g. AMM, EM, CMM, IPC, WBM, WDM/AWM, ELA, SPM, PMT, OSD)

**Weight & Balance Information**

| | | | |
|---|---|---|---|
| Weight Change : | Units | Position : | Units |

**Remarks**
- Proof of efficiency of disinfectants is not part of subject CRAS approval
- Evaluation of health risk for crew, passengers or ground operating staff is not part of subject CRAS approval

DISCLAIMER:
Deviating application of disinfectant:
When carrying out disinfection of an aircraft (e.g. at the special request of the client, or due to official or authority orders (intervention)), which deviates from the specifications set out by LHT in this document, unwanted changes may occur to the material surfaces. Among other things, this might negatively affect the visual appearance or functionality of the treated material.
Any subsequent actions to rectify this issue, such as replacement, repair or maintenance of the affected materials, are not the liability of the contractor or his subcontractor, but are the responsibility of the client.

| **Responsible for Design** | **Responsible for Design Verification** |
|---|---|
| Name / Dept. / Date / Signature | Name / Dept. / Date / Signature |
| Barth, Heiko FRAT/DS36<br>U437612<br>04.01.2023 11:53 | Signed by<br>Albert, Bernd<br>05.01.2023 09:55 |

**For classification and approval status refer to associated Change Repair Approval Sheet Project No. 00173859**

**Any use of this information being proprietary to Lufthansa Technik AG is prohibited unless approved in writing.**

**Accomplishment Instructions**

**1. Preparation / Material**

| No. | Qty | P/N | Description | Note |
|---|---|---|---|---|
| 01 | 2ea | | Minncare DryFogging Device | |
| 02 | a.r. | | Lerasept Spezial Dilution | max. 0,125% PAA concentration |
| 03 | a.r. | | Deionized Water | |
| 04 | 21ea | | Humidity/Temperature Sensors | wireless transmitting |
| 05 | 2ea | | Pressure Hose Spools | 5m |
| 06 | 2ea | | Air Compressor | for DryFog Device |
| 07 | 1ea | BM 1040167 | Air-Conditioning Ground Cart | or equivalent BM |
| 08 | 1ea | BM 1048148 | Air Supply Hose System (AirSnake) | or equivalent BM |
| 09 | a.r. | | Plastic Foil Covers | for Cabin/Galley area and Flight Deck Entrance |
| 10 | a.r. | SCOTCH-471-YELLOW-50MM Tape | | or equivalent. (For use on both external/internal A/C surfaces) |
| 11 | 2ea | | AIRLOCK DOOR | |
| 12 | a.r. | | Humidifier | remote controlled |

Info:

- Suitable Personal Protective Equipment (PPE) required for Disinfector and Maintenance Staff when accessing contaminated cabin.

**2. Accomplishment**

**THE FOLLOWING ACCOMPLISHMENT INSTRUCTIONS ARE FOR INITIAL TRIAL ON LHT OWNED A320 (A/C REG.: D-AOJT) ONLY!**

**Part A - Preparation of AircraftCabin:**

CAUTION: ENSURE ALL AIRCAFT SLIDES ARE SECURED AGAINST UNINTENDED DEPLOYMENT BEFORE BEGIN OF PREPARATION.

A01) Close all entrance and service doors except for DOOR 1L.
A02) Open all Overhead Stowages/ Cabinets / Lavatories / Galley compartments
 i. Remove one lavatory door in AFT cabin to improve spreading of dry fog.
A03) Fold down all tray table / in-arm tables.
A04) Remove all main cabin curtains.
A05) Remove all loose and emergency equipment as well as Safety Instructions Cards/Magazines from the Main Passenger Cabin Stowages.
 NOTE: Life Vests stowed in under-seat-stowages may remain on board.
A06) Remove loose catering equipment (e.g. Coffee Bowl) from Galleys
A07) Apply plastic foil around flight deck door and proper seal with suitable tape.
 NOTE: Install an “AIRLOCK” as an alternate option.
A08) Completely seal FWD Lavatory Door with suitable tape (min. width 50mm, e.g. Mat No. 10)
A09) Install a plastic foil/cover on all AFT Lavatory Smoke Detectors.
A10) Install “AIRLOCK” at DOOR 1L.
A11) Set Cabin Lighting to DIM MODE.
A12) Open all passenger overhead air outlets.


**Any use of this information being proprietary to Lufthansa Technik AG is prohibited unless approved in writing.**

**Part B – Setup of Dry Fogging Equipment in Aircraft Cabin:**

CAUTION: CONCENTRATED PAA CAN CAUSE SEVERE CORROSION TO AIRCRAFT STRUCTURE. THEREFORE HANDLING OF FLUID IS ONLY ALLOWED OUTSIDE OF AIRCRAFT.

CAUTION: SPRAY NOZZELS OF MINNCARE DEVICES MUST BE PROPERLY ALIGNED IN LONGITUDINAL AISLE DIRECTION.

B01) Prepare LERASEPT® SPEZIAL dilution and fill up fluid container of MINNCARE device outside of the aircraft.
B02) Position Minncare “01” at AFT CABIN position. Refer to attached FIGURE 01 for details.
B03) Position Minncare “02” at FWD CABIN position. Refer to attached FIGURE 01 for details.
B04) Position Air Compressor for each Minncare device and connect pressure hose. Refer to attached FIGURE 01 for details
NOTE: Route Power Supply Cord for Air Compressors through Door 1LH.
B05) Place a sheet of suitable absorbent paper under each Minncare device for monitoring of any fluid spill.
B06) Position Humidity/Temperature Sensors evenly throughout main cabin. Refer to attached FIGURE 02 for details.
NOTE: The exact positions (e.g. under the seat, inside shelf) shall be defined by the professional disinfector during the preparation, dependant from the cabin configuration.

**Part C – Pre-conditioning of Aircraft Cabin Environment**:

C01) Connect Air-Conditioning Ground Cart and prepare aircraft for external air-conditioning configuration i.a.w. AMM.
Ref. Task 12-33-21-618-001-A
“Pre-conditioning through the LP Ground Connection (Aircraft Electrical Power Available)”
or
Ref. Task 12-33-21-618-001-A03
”Pre-conditioning through the LP Ground Connection (Aircraft Electrical Power Not Available)”
Make sure that
- the airflow is not more than 78 kg/min (171.96 lb.min) equals 3600 $m^3$/h or 4680 kg/h.
- the supplied air pressure is not more than 40 mbar (0.58 psi) at the aircraft interface connector.
- the supplied air temperature does not increase to more than 70 DEG.C (158 DEG.F).

(OPTIONAL):
Ref. Task 12-33-21-618-001-A01
“Pre-conditioning through the HP Ground Connection”
Make sure that
- the ground air-supply unit generates an airflow between 54 kg.min (119.0496 lb.min) and 78 kg.min (171.9605lb.min)
- the supplied air temperature does not go above 260 deg.C (500.00 deg.F)

C02) If access to flight deck was necessary – re-apply/seal plastic foil (or close AIRLOCK) at flight deck entrance.
C03) Activate the wireless transmitting temperature and humidity sensors evenly distributed in the cabin to monitor the environmental conditions in real time (see step B06). Refer to FIGURE 02 for positions.

C04) IF NECESSARY - Install humidifier(s) (Mat No.12) evenly distributed in the cabin to control the air humidity in real time.
NOTE: The exact location(s) shall be defined by the professional disinfector during the preparation, dependant from the cabin configuration.


**Any use of this information being proprietary to Lufthansa Technik AG is prohibited unless approved in writing.**

C05) Start cabin preconditioning until environment parameters are levelled at about 18-20°C and 40-50% humidity. Once the parameters have levelled, switch off the Air-Conditioning Ground Cart.

C06) If required - Close/seal AIRLOCK at DOOR 1L.

C07) Maintain this environment for approx. 1- 2 hours BEFORE THE DISINFECTION STARTS to achieve equal surface temperatures of 20°C (+/- 2°C) and desired humidity throughout the cabin prior to start of disinfection process.

C08) In order to retain those conditions during the preconditioning, do the following:

- i.) In the first 30 minutes after the preconditioning has started, do a readout of all temperature and humidity sensors every 5 Minutes.
- ii.) When the value in regard exceeds the allowed range, activate the (LP or HP) Air-Conditioning Ground Cart i.a.w. related AMM TASK.
  NOTE: If humidity falls below target values, activate humidifier accordingly.
- iii.) After 30 minutes of test runtime, decide whether to increase or decrease the readout interval of temperature / humidity values taking into respect the then observed stability of the conditions.

**Part D – Application of Dry Fogging Disinfection in Aircraft Cabin**:

WARNING: ENTERING OF AIRCRAFT CABIN DURING ONGOING DISINFECTION SEQUENCE AND INITIALIZING OF VENTILATION IS ONLY ALLOWED WHEN EQUIPPED WITH PROPER PPE.

D01) Activate air compressors to start dry fogging with LERASEPT® SPEZIAL dilution.
NOTE: Ensure proper operation of Minncare devices by visual observation (e.g. through Side Windows or Camera)

D02) Monitor cabin humidity and ensure a level of 85%-95% is achieved at all sensor positions.
NOTE: Individually control fogging for each Minncare device as necessary.

D03) When all sensors show a humidity of >90%, stop air compressors / dry fogging.

- i) Record START TIME of DISINFECTION sequence.

NOTE: For proper disinfection of all surfaces, this environment (20°C (+/- 2°C) and >85% humidity) must be maintained for minimum of 3hours (180minutes)

D04) Maintain disinfection cabin environment for 3hours (180 minutes):

- i.) Re-adjust humidity when it drops below 85% by starting air compressor(s)/dry fogging again.
- ii.) When a humidity level of >90% is reached, stop air compressor(s)/dry fogging again.
- iii.) Repeat steps i) and ii) as necessary.
- iv.) Record END TIME of DISINFECTION sequence.
- v.) Deactivate all air compressors to stop dry fogging.

**Part E – Ventilation of Aircraft Cabin after Dry Fogging Disinfection:**

WARNING: ENTERING OF AIRCRAFT CABIN DIRECTLY AFTER END OF DISINFECTION SEQUENCE IS ONLY ALLOWED WHEN EQUIPPED WITH PROPER PPE.

ACCESS TO AIRCRAFT CABIN WITHOUT PPE DURING ONGOING VENTILATION IS ONLY ALLOWED WHEN MEASSURED H2O2 VALUES ARE BELOW 0,5PPM!

CAUTION: DO NOT ACTIVATE AIR CONDITIONING PACKS (E.G. PER APU) BEFORE 60 MINUTES OF VENTILATION.

E01) After completion of Dry Fogging Disinfection sequence, remove AIRLOCK from Door 1L and open all aircraft entrance doors (Door 1L / 4L) and service doors (Door 1R / 4R). Ventilate cabin for a minimum of 8 hours.



**Any use of this information being proprietary to Lufthansa Technik AG is prohibited unless approved in writing.**

i.) Install the Safety Barrier in the Passenger/Crew Door Frame as necessary. Refer to AMM SUBTASK 52-00-00-481-054-A

OPTIONAL: Support the ventilation and air drying process by operating the Low Pressure (LP) Air-Conditioning Ground Cart in the ventilation ("LÜFTEN") modus as possible. Refer to step C01).

E02) If possible – Intermittently operate the air conditioning packs as an alternate to LP Air-Conditioning Ground Cart for improved efficiency. Refer to step C01)

**Part F – Close-Up**

F01) Remove Minncare devices, pressure hoses and temperature/humidity sensor from cabin.
F02) Remove protective foil (or optional AIRLOCK) from flight deck entrance.
F03) Remove all protective foils/covers from Lavatory Smoke Detectors.
F04) Check cabin overhead stowage bins and doors for condensation droplets and clean/wipe off with a lint free cloth as necessary.
F05) Clean/wipe Galley food preparation counter tops with damp cloth.
F06) Fold up all seat tray tables and in-arm tables.
F07) Reinstall all loose and emergency equipment.
F08) Reinstall all loose catering equipment to Galleys.
F09) Reinstall main cabin curtains.
F10) Close all Overhead Stowages/ Cabinets / Lavatories / Galley compartments.
i. Reinstall lavatory door in AFT cabin.
F11) Disconnect ground equipment (e.g. Air Condition Ground Cart). Refer to AMM.
F12) Return aircraft back to serviceable condition.

**3. Adjustment / Tests**
- Perform operational test of Lavatory Smoke Detector per AMM Task 26-17-00-710-001-A.
- Perform operational check of the Cargo-Compartment Smoke Detection by CFDS per AMM Task 26-16-00-710-001-A
- Perform operational test of Passenger Announcement System per AMM Task 23-32-00-700-001-A.
- Perform functional test of Cabin Attendant Panel(s) per AMM Subtask 23-73-11-710-051-C.
- Perform functional test of Emergency Exit Lights per AMM TASK 33-51-00-710-001-A
- Perform functional test of PSU indications (FSB / NS Signs / Crew Call) per AMM Task 23-73-00-710-017-A.
- Please report any findings to PART 21J Engineering FRA T/DS36.



**Any use of this information being proprietary to Lufthansa Technik AG is prohibited unless approved in writing.**
